# Associations between Life’s Essential Eight cardiovascular health metrics and cardiovascular mortality risk across frailty statuses: evidence from a UK Biobank cohort study

**DOI:** 10.3389/fpubh.2025.1508274

**Published:** 2025-05-21

**Authors:** Lirong Chai, Kai Zhang, Yi Zhang, Weijing Wang, Dongfeng Zhang, Junning Fan

**Affiliations:** Department of Epidemiology and Health Statistics, School of Public Health, Qingdao University, Qingdao, China

**Keywords:** frailty, cardiovascular health, cardiovascular mortality risk, cohort study, survival analysis

## Abstract

**Background:**

Higher cardiovascular health (CVH) scores are related to lower risk of cardiovascular disease (CVD) mortality, and frailty status may moderate the association. Whether the associations of Life’s Essential 8 (LE8) with mortality from CVD and its subtypes differ across frailty status remains unknown. Therefore, we aimed to assess the association between LE8 and CVD mortality among individuals with different frailty status.

**Methods:**

Data were sourced from the UK Biobank of 439,462 participants aged 37–73 years. LE8, as a metric of CVH, was assessed using four health behaviors (diet, physical activity, nicotine exposure, and sleep health) and four health factors (body mass index, blood lipids, blood glucose, and blood pressure). Frailty status was measured with frailty index (FI) and Fried phenotype (FP). The outcomes included mortality of CVD, coronary heart disease, and cerebrovascular disease. Cox regression was used to calculate hazard ratios (HR) and 95% confidence intervals (CI) to assess the association, and additive and multiplicative interactive effects were also examined.

**Results:**

Over a median follow-up period of 13.7 [interquartile range 13.0–14.4] years, 6,085 participants died from CVD. The moderate or high level of LE8 lowered the risk of CVD mortality with HRs (95% CIs) of (0.50, 0.47–0.53) and (0.25, 0.22–0.29), respectively. The effect did not differ in individuals with different frailty status (*P_interaction_* > 0.05), each group with an HR of about 0.3. Compared with those with low LE8 and frail, the HR for individuals who are not frail and with high LE8 level was about 0.15. Similar results were found for endpoints of CVD subtypes and for participants of all ages and sexes, and specifically, CVH appeared to be better protected for CVD mortality in those who were not treated for blood pressure, cholesterol, and diabetes.

**Conclusion:**

Ideal CVH was associated with lower risk of CVD mortality regardless of frailty status. Specifically, for frail participants, optimizing CVH is a cost-effective strategy to mitigate CVD risk and promote healthy ageing.

## Introduction

Cardiovascular diseases (CVD), including ischemic heart disease and stroke, ranked as the first-leading cause of death, as reported in The Global Burden of Diseases, Injuries, and Risk Factors Study (GBD) 2021 publication (1). Therefore, studying the influencing factors of CVD mortality is one of the main directions of CVD research in the future (2). The American Heart Association has proposed *Life’s Essential 8* score (LE8) (3) as new metrics for Cardiovascular health (CVH) in 2022, which including four health factors (blood glucose, blood lipids, blood pressure, and body mass index (BMI)) and four health behaviors (smoking, sleep, physical activity, and diet). Compared to the Life’s Simple 7 (LS7) (4), the LE8 is enhanced by including sleep quality and an upgraded algorithm to quantify CVH. Some studies have shown that ideal CVH (higher LE8 component score) was effective in reducing the risk of CVD onset and mortality (5–7).

Frailty is an unstable state manifested as increased vulnerability to stressors, leading to higher risk of adverse outcomes including CVD death. Prevention of CVD in frail population remains to be an important issue, considering the rapid pace of aging (8) and the poorer prognosis of frailty groups (9). Existing studies found that frailty status is associated with the risk of CVD mortality, with participants in the pre-frail or frail status having a higher risk of CVD mortality compared to robust individuals (10–12). Of these, the frailty index (FI) and the frailty phenotype (FP) are currently the most common methods for evaluating frailty status. The definition of the FI is based on the Rockwood Cumulative Deficit Model (13, 14), which quantifies frailty by accumulating an individual’s health deficits. While FP is based on five core physiological function indicators (weight loss, exhaustion, low grip strength, low physical activity, and slow walking pace), reflecting the decline of multi-system physiological reserve (15). The two methods define frailty from different theoretical perspectives, reducing methodological bias and supporting the biological generalizability of the frailty-CVD association.

Some previous studies have found that ideal CVH scores can significantly reduce the risk of CVD mortality in specific sub-populations such as patients with hypertension (16), diabetes (17), and stroke (18), and reduce all-cause mortality or premature death in specific populations such as patients with chronic kidney disease (19) and without type 2 diabetes (20). However, whether the effect of CVH on CVD and its subgroups mortality differs or not among individuals of different frailty status remains quite unknown, which may provide clues for precise prevention of different populations. Meanwhile, there are significant differences in CVH status among people of different genders and ages (21). So, further research is needed to determine whether there is an interaction between frailty and CVH, and whether this effect is influenced by other factors such as age and sex.

Based on existing evidence, we hypothesized that higher LE8 scores will be associated with lower CVD mortality. The aims of this study were as follows: (1) to investigate the association between CVH and risk of CVD mortality; (2) to assess the variability of the above association by frailty status constructed from FI or FP; and (3) to perform the above analyses stratified by age and sex.

## Methods

### Study population

UK Biobank (UKB) is a large-scale prospective cohort study. At baseline survey from April 2006 to December 2010, 502,370 participants aged 37 to 73 years were recruited from the general population of the United Kingdom (UK). Sample information was collected by questionnaire, physical examination, and biological specimen monitoring at 22 assessment centers in England, Scotland, and Wales (22). Information on causes and dates of deaths were obtained from death certificates held by the National Health Service (NHS) Information Centre (England and Wales) and NHS Central Register (Scotland). UK Biobank has approval from the North West Multi-Centre Research Ethics Committee (MREC) (REC reference: 11/NW/03820) and written informed consent was obtained from all participants. Detailed information about the UK Biobank can be acquired at https://www.ukbiobank.ac.uk/ and previous publications (22).

In the current study, considering that individuals with prevalent CVDs at baseline may have altered health behaviors or conditions, we excluded participants with CVD diseases at baseline (*N* = 29,618). We also excluded pregnant women (*N* = 135), those with missing FP items (*N* = 32,761), and with ≥10 missing FI items at baseline (*N* = 394), finally, 439,462 participants were included in the main analyses, and the specific flow chart is shown in Supplementary Figure S1.

### Assessment of CVH

We used LE8 to assess CVH, which included four health behaviors (diet, physical activity, nicotine exposure, sleep health) and four health factors (body mass index, blood lipids, blood glucose, and blood pressure), each with a separate scoring algorithm ranging from 0 to 100. This allowed the generation of a new composite CVH score (an unweighted average of all components) that ranged from 0 to 100, with higher score indicating better CVH. In accordance with AHA recommendations and previous studies (23–25), we used the following categories to classify CVH status: 80 to 100 points (high, which deemed as ideal), 50 to 79 (moderate), 0 to 49 (low). The specific definitions of the indicators and field IDs were detailed in Supplementary Table S1.

### Assessment of frailty status

We constructed FI following standard procedures and used 49 items raised by previous researchers that constructed FI using data of UK Biobank (14, 26). We excluded three items that were related to CVD in FI, i.e., myocardial infarction, angina, and stroke, and eventually used 46 items to build FI. Participants with ≥ 10 missing items were excluded. For participants with missing items ≤ 9, FI was calculated as the sum of non-missing deficits a participant accumulated divided by the number of non-missing items. According to previous studies (12, 27, 28), the FI was further divided into three levels: robust (FI ≤ 0.10), pre-frail (0.10 < FI < 0.25), and frail (FI ≥ 0.25).

We constructed FP using five phenotypes, i.e., weight loss, exhaustion, low grip strength, low physical activity, and slow walking pace (15, 29). Detailed definitions of each criterion and field IDs are shown in Supplementary Table S2. Participants were classified as frail (3 to 5 phenotypes), pre-frail (1 to 2 phenotypes), or robust (0 phenotype) based on the number of criteria they met.

### Ascertainment of outcomes

In this study, we used mortality of CVD as the primary outcome and mortality of specific types of CVD including coronary heart disease (CHD) and cerebrovascular disease (CED) as secondary outcomes. The International Classification of Diseases, 10th revision (ICD-10) was used to define the following outcomes: CVD (I00-I99), CHD (I20-I25), CED (I60-I69). Date on deaths were available through November 30, 2022, and analyses were censored on that date or the date of death, whichever occurred first. Detailed information on the ascertainment of outcomes is available online at https://biobank.ctsu.ox.ac.uk/crystal/exinfo.cgi?src=Data_providers_and_dates.

### Covariates

The following covariates were selected by reference to previous studies (30, 31), mainly included demographic variables (age, sex, ethnicity, region, education, income, employment status, and Townsend Deprivation index), alcohol consumption, number of long-term medical conditions, and polypharmacy (i.e., current use of five or more medications). Details of covariates are provided in Supplementary Text S1.

### Statistical analyses

We used means (SD) and percentages to describe participants’ baseline characteristics across the three CVH categories. Differences between groups were analyzed by ANOVA for continuous variables and *χ*^2^ test for categorical variables.

We calculated crude mortality rate by dividing the number of events by person-years and used multivariable Cox proportional hazards regression to analyze hazard ratios (HRs) and 95% confidence intervals (CIs) of CVH categories with the mortality risk of CVD and its subtypes (CHD and CED). The proportional hazards (PH) assumption for the Cox model was checked using Schoenfeld residuals but not satisfied. However, even in the presence of nonproportionality, the Cox HR still provides a useful summary statistic to describe the average association between CVH and risk of CVD mortality during follow-up (32). We adjusted for possible confounders in three steps. Model 1 included age, sex, region, and ethnicity. Model 2 included model 1 plus education level, Townsend deprivation index, household income, and employment status. Model 3 included model 2 plus alcohol consumption. The missing values of the covariates were treated as dummy variables in the regression models. To assess whether the CVH-CVD association differed by frailty status, we investigated the association between CVH and the risk of CVD mortality in populations with different frailty conditions (robust, prefrail, and frail). The *p*-values for the multiplicative interactions were tested by likelihood ratio tests, and the relative excess risk due to interaction (RERI) were tested using the delta method (33) to assess the additive interaction of binary frailty and CVH on CVD mortality. Meanwhile, to evaluate the combined effect of frailty status and CVH with CVD mortality, we performed joint analyses by setting participants with frail and low CVH as the reference.

Considering the possible non-linear relationship between CVH and the risk of CVD mortality, we conducted restricted cubic spline (RCS) analyses with four knots (34, 35) (at the 5th, 35th, 65th and 95th percentiles) to examine the dose–response shapes between the continuous CVH score and the mortality risk of CVD and its subtypes by frailty status. We also assessed the associations of continuous health factors score and health behaviors score with CVD mortality by frailty status. Meanwhile, we also investigated the association of single CVH components with CVD mortality.

To explore whether the effect of CVH with CVD mortality differed by baseline characteristics, we examined the associations of categorical CVH metrics with the primary outcome of CVD mortality, stratified by age, sex, ethnicity, education, Townsend deprivation index, smoking status, alcohol consumption, physical condition, and number of long-term conditions, respectively. We further examined the association of frailty status measured with FI and FP with risk of CVD mortality. Meanwhile, we also examined the joint association of FI, FP and CVH with the risk of CVD mortality under stratification of sex, age, medical treatment of blood pressure, cholesterol and diabetes, and polypharmacy, respectively. We used likelihood ratio tests to test the statistical significance of differences between subgroups, considering Bonferroni adjustments. Considering the effect of different drug classes on the outcome, we sequentially adjusted for different drugs and polypharmacy based on model 3 to investigate their association with the risk of CVD death.

In sensitivity analyses, first, we further adjusted for the number of long-term conditions (model 4) based on model 3. Second, to minimize potential reverse causality, we excluded patients who had cancer at baseline or died for CVD within the first 2 years during follow-up, since participants with pre-existing cancer or early CVD-related deaths might already have underlying biological or lifestyle factors that significantly influence the outcomes being studied. Third, as an alternative approach to consider competing risks of mortality from other causes, we considered other-cause related deaths as a competing risk event for CVD mortality, and used Fines and Gray subdistribution risk models (36, 37) to calculate cumulative incidence functions (CIFs) in the primary analysis. Finally, considering that the main analysis did not satisfy the PH assumptions, we adopted time-stratified Cox models, introducing interaction terms of time groupings with age and gender, so that they satisfy the PH assumptions, and validating the main results.

Statistical significance was set at *p* < 0.05 (2-sided). All analyses were performed using Stata 15.0 (StataCorp, TX, United States) and R 4.3.1 (R Foundation) software.

## Results

Of 439,462 participants included in this cohort study, 6,085 died from CVD over a median follow-up period of 13.7 [IQR 13.0–14.4] years, including 2,786 deaths from CHD and 1,396 deaths from CED. In the current study, the mean (SD) age was 56.2 (8.1) years and 55.7% were women (Table 1). In general, individuals in the high cardiovascular health group were younger, tended to be female and white, and were also more likely to have higher educational attainment, have higher incomes and remain in the workforce, as well as have a lower Townsend deprivation index (i.e., less deprivation). Participants with high CVH scores were also more likely to be never smoker and physically active, to have a lower BMI and fewer long-term illnesses, and to have lower prevalence of frailty.

**Table 1:** Baseline characteristics of 439,462 participants in UK Biobank by cardiovascular health metrics.

Characteristics	All	Cardiovascular health metrics			*p* value
		Low (0–49)	Moderate (50–79)	High (80–100)	
Number	439,462	56,720	334,777	47,965	
Age (years)	56.2 (8.1)	57.5 (7.6)	56.5 (8.0)	52.4 (8.0)	<0.001
Women (%)	55.7	51.8	54.1	71.2	<0.001
Ethnicity (%)					<0.001
White	94.8	93.5	94.9	95.7	
South Asian	1.7	1.8	1.7	1.4	
East Asian	0.3	0.2	0.3	0.5	
Black	1.5	2.3	1.4	0.9	
Other/Mixed	1.4	1.7	1.4	1.4	
Unknown	0.3	0.4	0.3	0.2	
Educational level (%)					<0.001
College/University	33.8	19.4	33.9	49.7	
A/AS Levels/Equivalent	11.5	9.2	11.6	13.4	
Levels/GCSEs/Equivalent	21.5	21.6	21.8	18.9	
CSEs/Equivalent	5.4	6.5	5.4	4.4	
NVQ/HND/HNC/Equivalent	6.4	7.9	6.6	3.8	
Other professional qualifications	5.2	5.6	5.3	4.0	
None	15.4	28.2	14.7	5.4	
Income-to-poverty ratio (%)					<0.001
Less than £18,000	18.1	26.7	17.6	11.0	
£18,000 to £30,999	21.8	21.9	22.3	18.4	
£31,000 to £51,999	23.2	18.5	23.6	25.9	
£52,000 to £100,000	18.5	11.3	18.7	26.2	
Greater than £100,000	5.0	2.2	4.9	9.1	
Employment status (%)					<0.001
Working	59.7	51.7	59.2	72.4	
Retirement	31.9	34.5	33.2	19.3	
Other	8.2	13.3	7.3	8.1	
Townsend deprivation index	−1.4 (3.0)	−0.6 (3.3)	−1.5 (3.0)	−1.7 (2.8)	<0.001
Smoking status (%)					<0.001
Never smoker	55.6	30.3	56.9	76.4	
Ex-smoker	33.9	40.1	34.4	23.0	
Current smoker	10.2	28.9	8.4	0.4	
Alcohol intake frequency (%)					<0.001
Weekly	70.3	62.2	71.4	72.9	
Monthly	22.2	27.7	21.5	20.7	
Never	7.4	10.0	7.1	6.4	
Physical activity (%)					<0.001
Low	15.1	25.9	14.3	7.9	
Medium	33.7	18.5	35.2	41.0	
High	33.6	8.3	35.4	50.4	
BMI (kg/m^2^)	27.3 (4.7)	31.4 (5.6)	27.1 (4.3)	23.5 (2.6)	<0.001
No. of long-term conditions	1.1 (1.1)	1.6 (1.3)	1.1 (1.1)	0.7 (0.9)	<0.001
Frailty index (%)					<0.001
Robust	42.3	24.5	43.2	57.3	
Prefrail	50.6	57.8	50.8	40.6	
Frail	7.1	17.8	6.0	2.1	
Frailty phenotype (%)					<0.001
Robust	55.8	36.4	57.4	68.2	
Prefrail	40.3	51.3	39.8	31.0	
Frail	3.8	12.4	2.8	0.7	

BMI, body mass index; Values are expressed as mean (SD) or percentage. Analysis of variance was used for continuous variables and the chi-square test was used for categorical variables.

**Figure 1:**
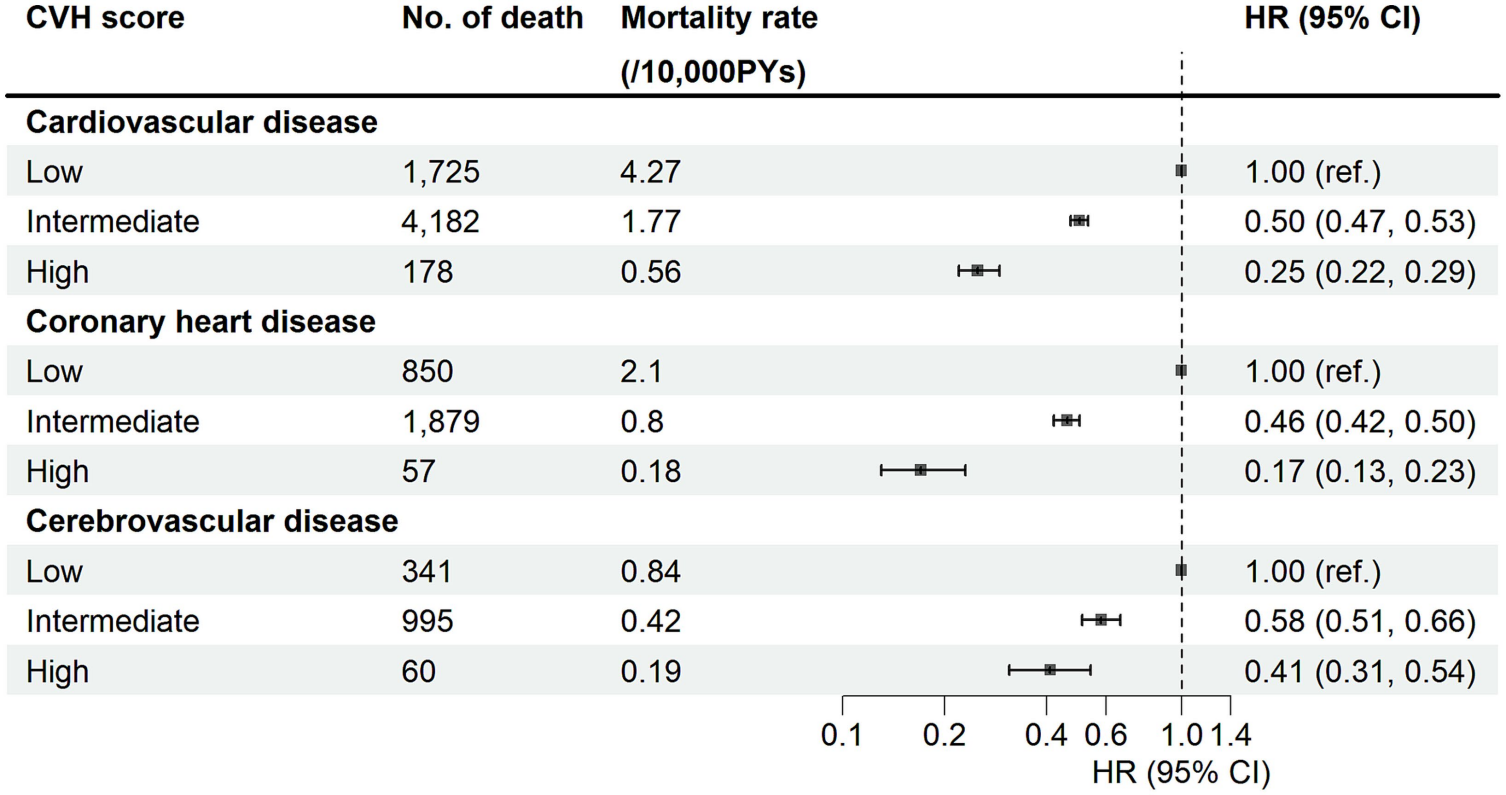
Association of cardiovascular health metrics and CVD mortality. CVH, cardiovascular health metrics; HR, hazard ratios; CI, confidence intervals; PYs, person-years. Cox regression model was adjusted for age, sex, region, ethnicity, education level, Townsend deprivation index, household income, employ status, and alcohol consumption.

Figure 1 depicted the association between categorical CVH and the risk of CVD mortality. The results showed a gradual decrease in the risk of mortality as the CVH score increased. The HRs (95% CIs) for the association of CVH with CVD mortality were 0.50 (0.47, 0.53) and 0.25 (0.22, 0.29) for the intermediate and high CVH groups, respectively, compared with the low CVH group. In the association of CVH with CHD and CED mortality, the HRs (95% CIs) of the high CVH group were 0.17 (0.13, 0.23) and 0.41 (0.31, 0. 54), respectively. Meanwhile, some of these single ideal CVH factors were also effective in reducing the risk of CVD mortality. Similar trends toward reduced risk of CVD mortality were observed for higher individual CVH scores of physical activity, nicotine, sleep health, and blood glucose (all *P* for trend < 0.05) (Supplementary Table S3). However, the scores for diet was not significantly associated with the risk of CVD mortality after fully adjusting for potential confounding factors. In contrast, the blood lipid score showed a positive association with CVD mortality (HR = 1.20, 95% CI: 1.13–1.28) in model 3. The results of RCS (Supplementary Figure S2) visualized the association of CVH and its components with the risk of CVD mortality, with results broadly consistent with Supplementary Table S2, it showed a J-shape association between blood lipids and CVD mortality.

**Figure 2:**
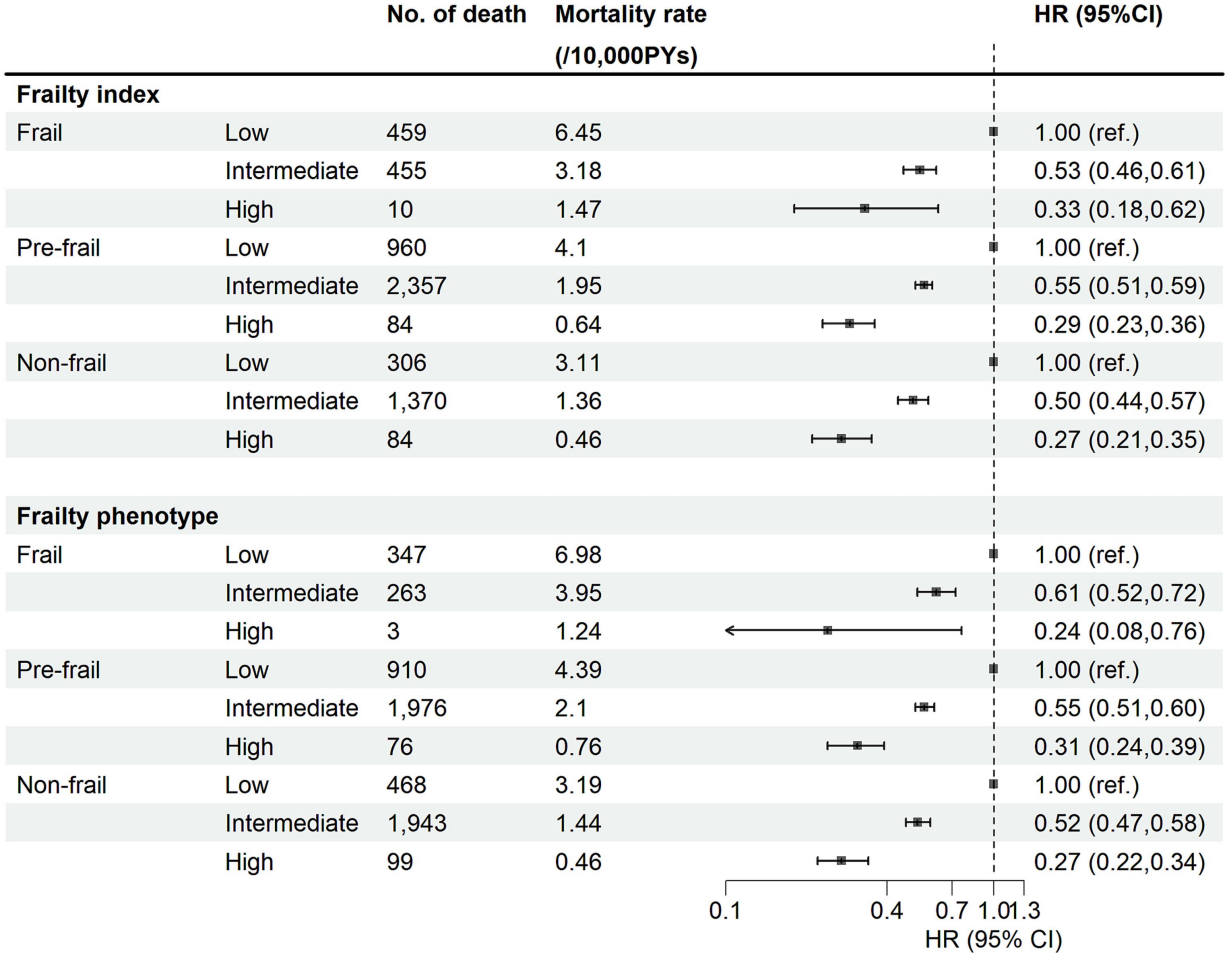
Association between cardiovascular health metrics and risk of CVD mortality by frailty status. HR, hazard ratios; CI, confidence intervals; PYs, person-years. Cox regression model was adjusted for age, sex, region, ethnicity, education level, Townsend deprivation index, household income, employ status, and alcohol consumption. The *p*-values for multiplicative interaction of frailty index and frailty phenotype were 0.863 and 0.401, the *p*-values for additive interaction were 0.011 (RERI = 0.397) and 0.203.

**Figure 3:**
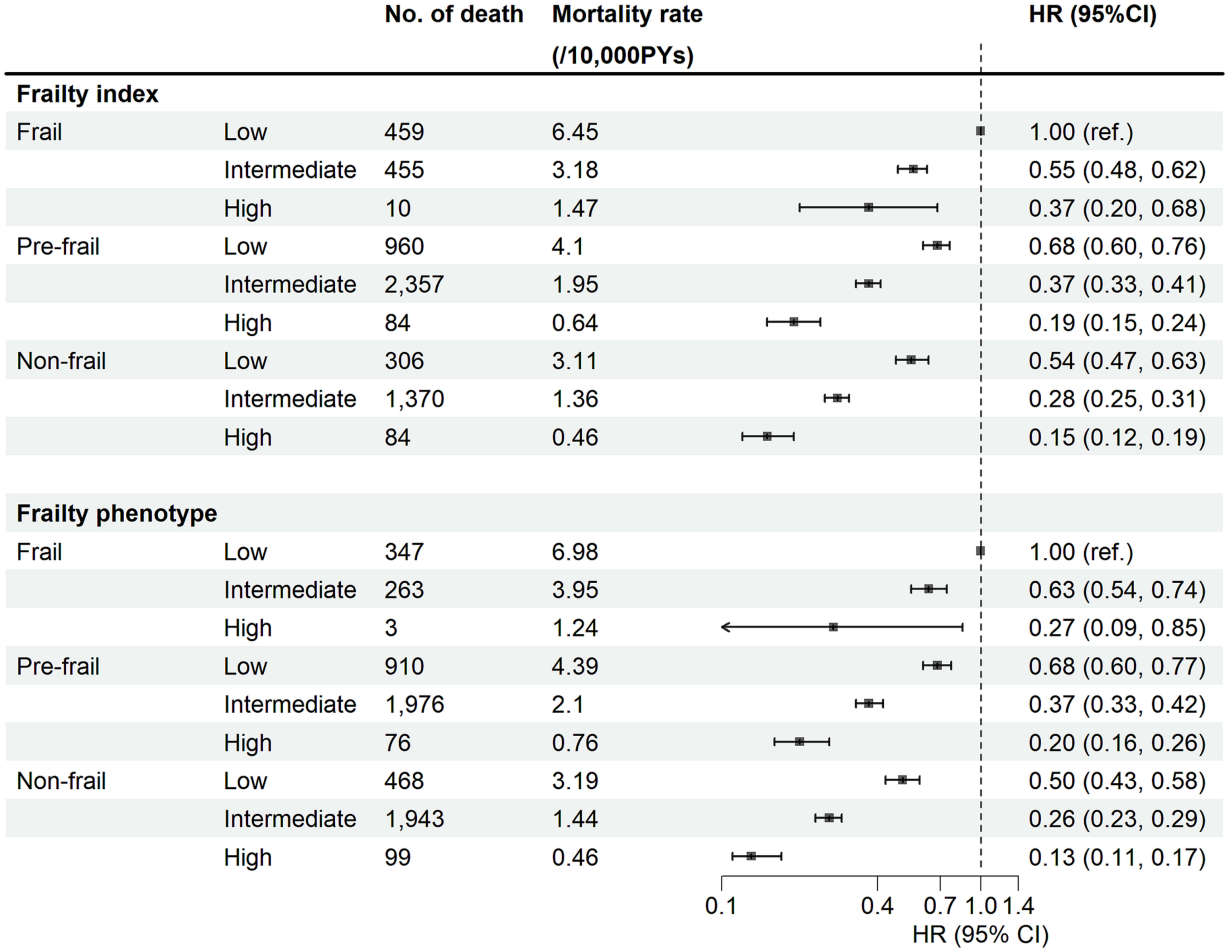
Joint association of frailty status and cardiovascular health metrics with risk of CVD mortality. HR, hazard ratios; CI, confidence intervals; PYs, person-years. Cox regression model was adjusted for age, sex, region, ethnicity, education level, Townsend deprivation index, household income, employ status, and alcohol consumption.

The associations between categorical CVH and the risk of CVD mortality by frailty status were presented in Figure 2. The reduction in the risk of CVD mortality by high CVH was consistently found in different frailty groups, and there was no multiplicative interaction between CVH and frailty in either FP or FI. However, in FI we found a co-multiplicative interaction between CVH and the risk of CVD mortality (RERI = 0.397, *P_interaction_* = 0.011), which was not present in FP (*P_interaction_* > 0.05). The interaction plots also indicated that variations in frailty status might influence the impact of CVH on CVD mortality risk (Supplementary Figure S3). When we used FI to measure frailty, the HRs for the high CVH in the frailty, pre-frailty, and healthy groups were 0.33, 0.29, and 0.27, respectively, and the HR for the intermediate CVH group similarly ranged from 0.50 to 0.55. When we used FP to measure frailty, the HRs for the high CVH in the frailty, pre-frailty, and healthy groups were 0.24, 0.31, and 0.27, respectively, and the HR for the intermediate CVH group similarly ranged from 0.52 to 0.61. In joint analyses (Figure 3), compared with participants with low CVH and frail, the strongest protective effect on the risk of CVD mortality was found in the non-frail and high CVH group, with the HR (95% CI) being 0.15 (0.12, 0.19) for FI and 0.13 (0.11, 0.17) for FP. Similar results were observed for CHD and CED mortality (Supplementary Figures S4–S7). Compared with individuals with frail and low CVH scores, those with non-frail and ideal CVH scores had the lowest risk of mortality.

**Figure 4:**
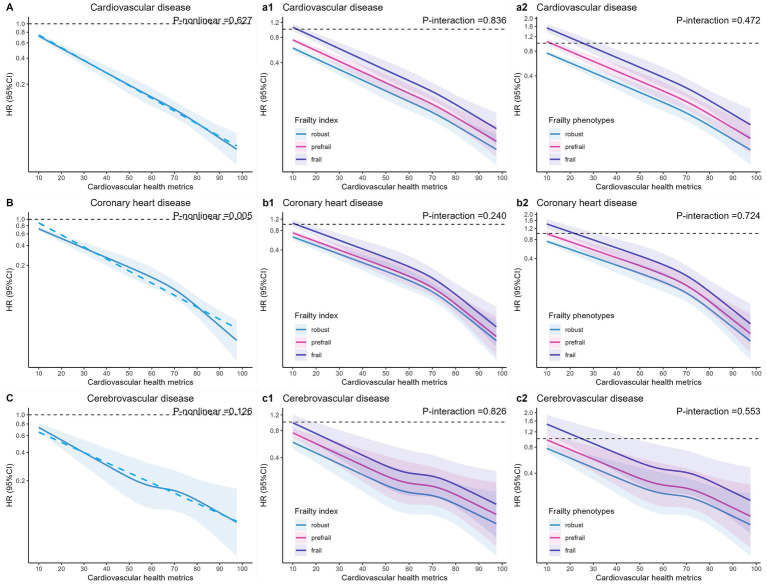
The hazard ratios (solid line) and 95% confidence intervals (band) were estimated by fitting restricted cubic spline Cox regression models with 5th, 35th, 65th and 95th knots, in which cardiovascular health metrics was modeled as a continuous variable. The minimum value (cardiovascular health metrics =0) was set as the reference. Cox regression model was adjusted for age, sex, region, ethnicity, education level, Townsend deprivation index, household income, employ status, and alcohol consumption. **(A)** cardiovascular health metrics (CVH) and CVD mortality. **(a1)** CVH and CVD mortality by frailty index (FI). **(a2)** CVH and CVD mortality by frailty phenotypes (FP). **(B)** CVH and coronary heart disease (CHD) mortality. **(b1)** CVH and CHD mortality by FI. **(b2)** CVH and CHD mortality by FP. **(C)** CVH and cerebrovascular disease (CED) mortality. **(c1)** CVH and CED mortality by FI. **(c2)** CVH and CED mortality by FP.

Meanwhile, restricted cubic spline plots showed a significant linear negative association between continuous CVH and the risk of CVD and CED mortality (Figure 4), with the risk of mortality decreasing as the CVH score increased, whereas it showed a nonlinear (*P*-nonlinear = 0.005) association between CVH and the risk of CHD mortality. The above-mentioned associations were consistently found across different frailty status (*P_interaction_* > 0.05 for all). Significant linear negative associations were also found across the board between healthy behaviors (Supplementary Figure S8) and healthy factors (Supplementary Figure S9) and the mortality risk for CVD and its subtypes.

Stratified analyses showed that the association between CVH and risk of CVD mortality remained significant under baseline characteristic stratification, and the protective effect of CVH was stronger in younger individuals, women and those without polypharmacy (Supplementary Table S4, *P_interaction_* < 0.05). We also validated the increased risk of CVD mortality in both pre-frailty and frailty groups compared with non-frailty groups when using FI and FP (Supplementary Figure S10). Stratified analysis and joint association of frailty status and CVH with risk of CVD mortality showed similar results with the primary findings when stratified by age, sex, medical treatment of blood pressure, cholesterol and diabetes, and polypharmacy (Supplementary Figures S11–S20). Among these, CVH seems to be more protective of CVD morality among population without medical treatment of blood pressure, cholesterol and diabetes (Supplementary Figure S15, *P_interaction_* for FI = 0.005; *P_interaction_* for FP = 0.009). The associations between CVH and risk of CVD death after adjusting for different types of medical treatments were generally consistent with the main model (Supplementary Table S5). The results of the sensitivity analyses were broadly consistent with the main results (Supplementary Table S6), despite a slight decrease of the effect size after adjusting for long-term conditions.

## Discussion

This study investigated the association of CVH with mortality of CVD and its subtypes in a large cohort study. We found that moderate and ideal CVH was associated with lower risk of CVD mortality and there was a basically negative linear trend between CVH scores and CVD mortality, the protective effect of CVH was meaningful and broadly consistent across different levels of frailty regardless of using FI or FP. Taken together, compared with participants being frail and with low CVH level, individuals who are not frail and with ideal CVH level had 85% lower risk of CVD mortality. Results were similar across age and sex, but ideal CVH was more protective of the risk of CVD mortality in those not treated with blood pressure, lipid, and diabetes medications.

We found that a ideal CVH score can effectively reduce the risk of CVD mortality, and the mortality risk gradually decreased with the increment of CVH score, which is consistent with previous studies. Jiahong Sun et al. (6) included 19,951 US adults from National Health and Nutrition Examination Survey (NHANES) and found that compared with low CVH scores, moderate and ideal CVH scores were associated with 38% (HR = 0.62, 95% CI: 0.46–0.83) and 64% (0.36, 0.21–0.59) reduction in CVD mortality risk, respectively. A previous UKB study of 254,783 participants also found that ideal CVH was associated with lower risk of CVD mortality, with HR (95% CI) of 0.51 (0.46, 0.56) for intermediate CVH score and 0.27 (0.23, 0.32) for ideal CVH score compared with low CVH score (7). Meanwhile, we also found essentially negative linear associations between CVH, health behaviors and health factors with the mortality risk of overall CVD and subtypes.

Our study demonstrated that single ideal CVH factors (including physical activity, nicotine exposure, sleep health, blood glucose, and blood pressure) were also effective in reducing the risk of CVD mortality, with the strongest association being tobacco exposure, which reduced the risk by 59% (HR = 0.41, 95% CI: 0.39–0.44), followed by blood glucose, which reduced the risk by 57% (0.43, 0.40–0.47). A study based on NHANES similarly found (38) that physical activity, nicotine exposure, sleep health, BMI, blood glucose, and blood pressure were significantly related to cardiovascular mortality risk. The importance of ideal blood glucose was also found in another NHANES study (6). Rather strangely, our study found that moderate level of blood lipids reduced the risk of CVD mortality, but high level of blood lipids increased the risk of mortality. However, Jiahong Sun (6) and Jiayi Yi (38) both found no association between ideal blood glucose and CVD mortality. The possible reason may be the nonlinear relationship between lipids and CVD mortality risk, according to a longitudinal study (39) that included 12,574 individuals, it showed a U-shaped correlation between non-HDL and the risk of CVD mortality with a threshold value of 142 mg/dL.

The impact of CVH on health outcomes may vary in populations with diverse health statuses; therefore, exploring the association between CVH and CVD mortality in different sub-populations may provide evidence for precision prevention and intervention. Previous researchers have shown that, higher CVH was effective in reducing the risk of CVD mortality in the hypertensive participants of NHANES in 2007–2016 by 46% (HR = 0.54, 95% CI: 0.31–0.94) (16), in the type 2 diabetes group of UKB by 51% (HR = 0.49, 95% CI: 0.29–0.81) (17), and in stroke patients of NHANES in 2007–2018 by 49% (HR = 0.51, 95% CI: 0.26–0.98) (18). However, few studies have examined whether the CVH-CVD association was consistent in people of different frailty status. Our study supplement previous studies by shown that, maintaining an ideal CVH, regardless of baseline frailty status, reduced the risk of death from CVD by 59–83% for FI and 57–74% for FP, underscoring the importance to keep ideal cardiovascular habits to decrease premature mortality risk. Thus, for frail individuals, maintaining good cardiovascular health habits can be seen as a low-cost and feasible way to reduce CVD mortality. Of course, the same is true for robust individuals. Further, incorporating LE8 into clinical practice provides a comprehensive framework for assessing cardiovascular risk, especially in frail individuals.

Meanwhile, our study further found that maintaining non-frail as well as ideal CVH simultaneously reduced the risk of CVD mortality by 85%. A previous study included 314,093 participants from UKB, the authors found that physical frailty (FP) and Life’s Simple 7 were jointly associated with incident CVD (*P* for additive interaction <0.001), the participants of frailty accompanied poor CVH had the highest risk with HR of 2.92 (95% CI: 2.68–3.18) (31). Another study including 35,207 participants with 8.1 years of follow-up found (40) that both frailty and poor CVH increased the risk of CVD mortality, and this combined burden differed by age only in men, with a greater burden in older men. Meanwhile, Ning Ning et al. (30) found that participants in the low CVH and frail status group had a significantly increased risk of cardiovascular disease mortality compared with participants in the ideal CVH and non-frail status group (HR = 6.57, 95% CI: 3.54–12.22), based on the NHANES with 87 months of follow-up, which was more pronounced in young and women.

Meanwhile, there was a synergistic additive interaction between FI and CVH (RERI = 0.379), which indicated that the effect of frail status scored by FI with low CVH on the risk of CVD mortality was partly attributable to the additive interaction between the two, i.e., the interaction between the two enhances the risk of CVD mortality, and therefore, it is more important to pay attention to maintaining cardiovascular fitness and avoiding the co-existence of the two risk factors in people with frailty. In addition, although the interaction analysis showed no interaction between frail status scored by FP and CVH scores with the risk of CVD mortality, we still speculate that this may be related to confounders or analytic bias, and that the associations between the three should be further analyzed in larger cohorts in the future. Meanwhile, the difference in the effects of FI and FP on the association between CVH and CVD mortality risk may also stem from the difference in the methods of constructing the frail state between the two metrics, with FI quantifying frailty by accumulating health deficits in an individual and FP reflecting a decline in the functioning of the multi-system physiological reserve based on five core physiological functioning metrics. The related mechanisms need to be further investigated in the future. Our study using the updated LE8 complemented existing research that the combined impact of frailty and CVH on CVD mortality were consistently found in different ages, sexes and polypharmacy statuses. That is, maintaining ideal CVH levels, most of which were modifiable, can benefit individuals of all ages, sexes and polypharmacy statuses. Meanwhile, among those who were not treated with blood pressure, lipid and diabetes medications, the risk of death was lower in those with ideal CVH scores compared to those with low CVH, which may suggest that maintaining an ideal CVH in healthy populations who are not applying medications for the treatment of chronic diseases better reduces the risk of death from CVD. However, the results were not significant in separate drug treatments and polypharmacy, so further studies are needed in the future.

Our study thoroughly examined the association between CVH and CVD mortality in populations of different frailty status, suggesting the benefit of ideal CVH in people of different frailty grades, which may provide useful guidance for CVD prevention in frail people. Based on the results of this study, we believe that future prospective studies could stratify by treatment class or design frailty-specific trials of CVH interventions to assess differential effectiveness. Longitudinal research is also needed to clarify the paradoxical associations between lipid scores and CVD mortality, particularly in frail populations where treatment effects may be confounded. Implementation studies could further explore how LE8 might be incorporated into routine geriatric care to guide risk reduction strategies. Finally, replication of findings in more clinically diverse, older, and globally representative populations is essential to ensure generalizability.

However, our study still has some limitations. First, CVH metrics were mainly obtained through self-report and physical examination, and there may be information bias and measurement error. Nevertheless, health behaviors were collected using a standardized questionnaire with clear and accurate entries, and health factor measures were collected by trained professionals or medical staff to minimize information bias. Second, we excluded participants with missing values of FI or FP and participants with CVD at baseline, which may introduce a selection bias. Finally, despite careful adjustment for possible confounders, unmeasured or residual confounders may remain, such as cognitive status, self-care, etc.

## Conclusion

Our study highlights the importance of maintaining modifiable healthy cardiovascular habits, even in frail participants, to achieve cardiovascular fitness. We strongly recommend that residents can maintain ideal CVH and non-frail physical condition by means of a sensible diet, active physical activity, healthy sleep, and maintaining normal BMI, blood pressure, blood lipids, and blood glucose to minimize the risk of cardiovascular mortality, especially for those who are not treated for blood pressure, blood lipids, and diabetes medicine.

## Data Availability

The datasets presented in this article are not readily available because the researcher does not have permission to share the dataset. Requests to access the datasets should be directed to https://www.ukbiobank.ac.uk/.
