# Supplementary material for: Associations between Life’s Essential Eight cardiovascular health metrics and cardiovascular mortality risk across frailty statuses: evidence from a UK Biobank cohort study
Source: Front Public Health. 2025 May 21;13:1508274. doi: 10.3389/fpubh.2025.1508274 (PMC12133463; doi:10.3389/fpubh.2025.1508274)
Supplement: Supplementary file 1 [file Supplementary_file_1.docx]

**Supplementary appendix**

**Figure S1** Flow chart of inclusion and exclusion of participants.

**Table S1** New and Updated Metrics for Measurement and Quantitative Assessment of CVH.

**Table S2** Frailty criteria.

**Text S1** Detailed covariates.

**Table S3** Adjusted hazard ratios of Life’s Essential 8 cardiovascular health (CVH) components with risk of CVD mortality.

**Figure S2** Association between cardiovascular health metrics components and CVD mortality in cox regression with restricted cubic spline

**Figure S3** Interaction plots of the association of frailty status and cardiovascular health with the risk of mortality from cardiovascular and cerebrovascular disease

**Figure S4** Association between cardiovascular health metrics and risk of CHD mortality by frailty status.

**Figure S5** Association between cardiovascular health metrics and risk of cerebrovascular disease mortality by frailty status.

**Figure S6** Joint association of frailty status and cardiovascular health metrics with mortality from CHD.

**Figure S7** Joint association of frailty status and cardiovascular health metrics with mortality from cerebrovascular disease.

**Figure S8** Association between health behaviors metrics and CVD mortality by frailty status in cox regression with restricted cubic spline.

**Figure S9** Association between health factors metrics and CVD mortality by frailty status in cox regression with restricted cubic spline.

**Figure S10** Association of frailty index and frailty phenotype with CVD mortality.

**Table S4** Association between cardiovascular health metrics and CVD mortality by Subgroups.

**Figure S11** Joint association of frailty status and cardiovascular health metrics with risk of CVD mortality by gender.

**Figure S12** Joint association of frailty status and cardiovascular health metrics with risk of CVD mortality by age.

**Figure S13** Association between cardiovascular health indicators and risk of cardiovascular disease mortality by frailty status with gender stratification.

**Figure S14** Association between cardiovascular health indicators and risk of cardiovascular disease mortality by frailty status with age stratification.

**Table S4** Association of cardiovascular health metrics with CVD mortality adjusted for different medications

**Figure S15** Association between cardiovascular health indicators and risk of cardiovascular disease mortality by frailty status with medical treatment of blood pressure, cholesterol and diabetes stratification.

**Figure S16** Association between cardiovascular health indicators and risk of CHD mortality by frailty status with medical treatment of blood pressure, cholesterol and diabetes stratification.

**Figure S17** Association between cardiovascular health indicators and risk of cerebrovascular disease by frailty status with medical treatment of blood pressure, cholesterol and diabetes stratification.

**Figure S18** Association between cardiovascular health indicators and risk of cardiovascular disease mortality by frailty status with polypharmacy stratification.

**Figure S19** Association between cardiovascular health indicators and risk of CHD mortality by frailty status with polypharmacy stratification.

**Figure S20** Association between cardiovascular health indicators and risk of cerebrovascular disease mortality by frailty status with polypharmacy stratification.

**Table S5** Association of cardiovascular health metrics with CVD mortality adjusted for different medical treatments.

**Table S6** Sensitivity analyses association of cardiovascular health metrics with CVD mortality.

**
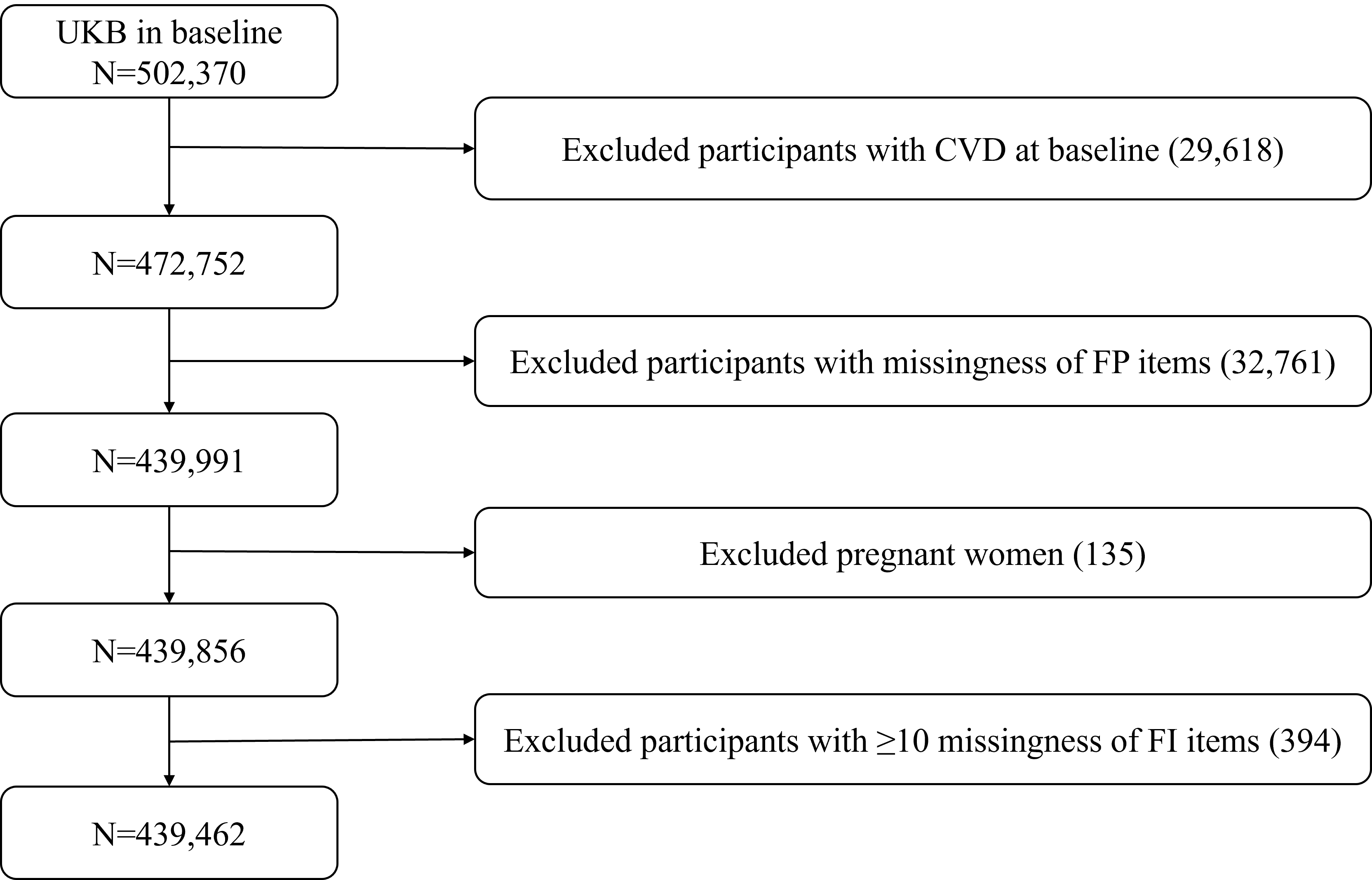
**

**Figure S1 Flow chart of inclusion and exclusion of participants**

FI, frailty index; FP, frailty phenotype.

**Table S1 New and Updated Metrics for Measurement and Quantitative Assessment of CVH**

| Domain | CVH metric | Definitions^[1]^ | Field ids |
| --- | --- | --- | --- |
| Health behaviors | Diet^[2, 3]^ | Self-reported daily intake of a DASH-style eating pattern modified according to available diet variables in UKB  Points: Modified DASH score  100 8-10  80 6-7  50 4-5  25 2-3  0 0-1 | 1309, 1319,1289, 1299,1438, 1448, 1458, 1468, 1428, 2654, 1438, 1329, 1339, 1408, 1418, 1438, 1448, 1458, 1468, 1359, 1369, 1379, 1389, 1349, 6144 |
|  | PA | Metric: Minutes of moderate- (or greater) intensity activity per week  Scoring:  Points Minutes  100 ≥150  90 120–149  80 90–119  60 60–89  40 30–59  20 1–29  0 0 | 22038, 22039 |
|  | Nicotine exposure | Metric: Combustible tobacco uses or inhaled NDS use; or secondhand smoke exposure  Scoring:  Points Status  100 Never smoker  75 Former smoker, quit ≥5 y  50 Former smoker, quit 1–<5 y  25 Former smoker, quit <1 y, or currently using inhaled NDS  0 Current smoker  Subtract 20 points (unless score is 0) for living with active indoor smoker in home | 20116, 1259, 2897 |
|  | Sleep health | Metric: Average hours of sleep per night  Scoring:  Points Level  100 7–<9  90 9–<10  70 6–<7  40 5–<6 or ≥10  20 4–<5  0 <4 | 1160 |
| Health factors | BMI | Metric: BMI (kg/m^2^)  Scoring:  Points Level  100 <25  70 25.0–29.9  30 30.0–34.9  15 35.0–39.9  0 ≥40.0 | 21001 |
|  | Blood lipids | Metric: Non–HDL cholesterol (mg/dL)  Scoring:  Points Level  100 <130  60 130–159  40 160–189  20 190–219  0 ≥220  If drug-treated level, subtract 20 points | 6153, 6177, 30690, 30760 |
|  | Blood glucose | Metric: FBG (mg/dL) or HbA1c (%)  Scoring:  Points Level  100 No history of diabetes and FBG <100 (or HbA1c <5.7)  60 No diabetes and FBG 100–125 (or HbA1c 5.7–6.4) (prediabetes)  40 Diabetes with HbA1c <7.0  30 Diabetes with HbA1c 7.0–7.9  20 Diabetes with HbA1c 8.0–8.9  10 Diabetes with Hb A1c 9.0–9.9  0 Diabetes^*^ with HbA1c ≥10.0 | 6153, 6177, 2443, 20002, 30750, 30740 |
|  | BP | Metric: Systolic and diastolic BPs (mm Hg)  Scoring:  Points Level  100 <120/<80 (optimal)  75 120–129/<80 (elevated)  50 130–139 or 80–89 (stage 1 hypertension)  25 140–159 or 90–99  0 ≥160 or ≥100  Subtract 20 points if treated level | 4079, 4080, 93, 94, 6153, 6177 |

Abbreviations: BMI indicates body mass index; BP, blood pressure; CVH, cardiovascular health; DASH, Dietary Approaches to Stop Hypertension; FBG, fasting blood glucose; HbA1c, hemoglobin A1c; HDL, high-density lipoprotein; NDS, nicotine-delivery system; PA, physical activity. ^*^Diabetes was diagnosed by a random blood glucose level ≥11.1 mmol/L, or a glycosylated hemoglobin (HbA1c) level ≥48 mmol/L (6.5%), or self-reported diabetes by questionnaire, or use of medication for diabetes treatment (insulin), or diagnosis of diabetes by physician's interview.

1. Lloyd-Jones DM, Allen NB, Anderson CAM, Black T, Brewer LC, Foraker RE, et al. Life's Essential 8: Updating and Enhancing the American Heart Association's Construct of Cardiovascular Health: A Presidential Advisory From the American Heart Association. Circulation. 2022;146(5):e18-e43.

2. Sun Y, Yu Y, Zhang K, Yu B, Yu Y, Wang Y, et al. Association between Life's Essential 8 score and risk of premature mortality in people with and without type 2 diabetes: A prospective cohort study. Diabetes/metabolism research and reviews. 2023;39(5):e3636.

3. Zhang J, Chen G, Xia H, Wang X, Wang C, Cai M, et al. Associations of Life's Essential 8 and fine particulate matter pollution with the incidence of atrial fibrillation. Journal of hazardous materials. 2023;459:132114.

**Table S2 Frailty criteria**

| Fried frailty phenotype | | Definitions | Field ids |
| --- | --- | --- | --- |
| Weight loss | Self-reported: “In the last year, have you lost more than 10 pounds unintentionally?” (response: yes=1, no=0) | | 2306 |
| Exhaustion | Self-reported (CES depression scale, two questions): “How often in the last week (a) did you feel that everything was an effort, or (b) could you not get going?” (response: moderate amount of the time [3–4 days] or most of the time=1, other=0) | | 2080 |
| Low physical activity | Self-reported: Minnesota Leisure Time Activity Questionnaire (18 items). Kcal of activity per week estimated, and the lowest 20% were identified as meeting frail criteria | | 6164 |
| Slow walking pace | Measured time to walk 15 feet | | 924 |
| Low grip strength | Measured grip strength, adjusted for sex and body-mass index (lowest 20% of cohort identified as meeting frail criteria) | | 21001, 31  46, 47 |

**Text S1**

Regions included England, Scotland, and Wales. Ethnicities included white, south Asia, east Asia, black, other/mixed, and unknown (0.3% missing data). Education levels included College or University degree, General Certificate of Education Advanced Level / Advanced Subsidiary level or equivalent, Singapore-Cambridge General Certificate of Education Ordinary Level / General Certificate of Secondary Education or equivalent, Certificate of Secondary Education or equivalent, National Vocational Qualification or Higher National Diploma or Higher National Certificate or equivalent, Other professional qualifications, none of the above, prefer not to answer (0.8% missing data). Total annual household income included less than £18,000, £18,000 to £30,999, £31,000 to £51,999, £52,000 to £100,000, greater than £100,000, do not know, prefer not to answer (13.4% missing data). Employment status was classified into four categories, working, retired, other, and missing (0.4% missing data). We categorized alcohol consumption into weekly, monthly, never and missing (0.05% missing data). Definition and list of long-term conditions were diagnosed by doctors recorded by nurse-led interview, except for cancer diagnosis which was reported by touch-screen questionnaire. The list of disease groupings was based on Barnett et al (2012)^[4]^. Medication use was obtained from nurse-led verbal interviews and questionnaires and included medications prescribed for chronic conditions, daily medications, and vitamins, minerals, and other dietary supplements. We used the most common definition of polypharmacy, which counted all concurrent medications and defined the use of ≥5 medications as polypharmacy^[5]^.

4. Barnett K, Mercer SW, Norbury M, Watt G, Wyke S, Guthrie B. Epidemiology of multimorbidity and implications for health care, research, and medical education: a cross-sectional study. Lancet (London, England). 2012;380(9836):37-43.

5. Chen LJ, Sha S, Brenner H, Schöttker B. Longitudinal associations of polypharmacy and frailty with major cardiovascular events and mortality among more than half a million middle-aged participants of the UK Biobank. Maturitas. 2024;185:107998.

**Table S3 Adjusted hazard ratios of Life’s Essential 8 cardiovascular health (CVH) components with risk of CVD mortality**

| CVH components | CVH score, HR (95% CI) | | | | *P* for trend |
| --- | --- | --- | --- | --- | --- |
|  | Low (0-49) | | Moderate (50-79) | High (80-100) |  |
| Total |  |  | |  |  |
| Mortality (/10,000PYs) | 4.27 | 1.77 | | 0.56 |  |
| Model 1 | 1.00 (ref.) | **0.42 (0.40, 0.45)** | | **0.20 (0.17, 0.24)** | <0.001 |
| Fully adjusted | 1.00 (ref.) | **0.50 (0.47, 0.53)** | | **0.25 (0.22, 0.29)** | <0.001 |
| Diet |  |  | |  |  |
| Mortality (/10,000PYs) | 2.06 | 1.87 | | 1.93 |  |
| Model 1 | 1.00 (ref.) | **0.91 (0.86, 0.96)** | | **0.88 (0.79, 0.98)** | <0.001 |
| Fully adjusted | 1.00 (ref.) | 0.95 (0.90, 1.00) | | 0.90 (0.81, 1.01) | 0.014 |
| Physical activity |  |  | |  |  |
| Mortality (/10,000PYs) | 2.40 | 1.93 | | 1.79 |  |
| Model 1 | 1.00 (ref.) | 0.85 (0.71, 1.01) | | **0.71 (0.67, 0.75)** | <0.001 |
| Fully adjusted | 1.00 (ref.) | 0.97 (0.81, 1.16) | | **0.79 (0.75, 0.83)** | <0.001 |
| Nicotine exposure |  |  | |  |  |
| Mortality (/10,000PYs) | 3.94 | 2.43 | | 1.45 |  |
| Model 1 | 1.00 (ref.) | **0.44 (0.41, 0.47)** | | **0.34 (0.32, 0.37)** | <0.001 |
| Fully adjusted | 1.00 (ref.) | **0.52 (0.48, 0.56)** | | **0.41 (0.39, 0.44)** | 0.002 |
| Sleep health |  |  | |  |  |
| Mortality (/10,000PYs) | 3.28 | 2.06 | | 1.82 |  |
| Model 1 | 1.00 (ref.) | **0.64 (0.58, 0.70)** | | **0.53 (0.49, 0.57)** | <0.001 |
| Fully adjusted | 1.00 (ref.) | **0.78 (0.71, 0.85)** | | **0.67 (0.62, 0.73)** | <0.001 |
| Body mass index |  |  | |  |  |
| Mortality (/10,000PYs) | 2.88 | 1.91 | | 1.41 |  |
| Model 1 | 1.00 (ref.) | **0.60 (0.56, 0.63)** | | **0.55 (0.51, 0.59)** | 0.139 |
| Fully adjusted | 1.00 (ref.) | **0.66 (0.62, 0.70)** | | **0.62 (0.57, 0.66)** | 0.148 |
| Blood lipids (non-HDL cholesterol) |  |  | |  |  |
| Mortality (/10,000PYs) | 1.98 | 1.50 | | 2.51 |  |
| Model 1 | 1.00 (ref.) | **0.86 (0.80, 0.93)** | | 1.25 (1.17, 1.33) | 0.862 |
| Fully adjusted | 1.00 (ref.) | **0.88 (0.82, 0.95)** | | **1.20 (1.13, 1.28)** | 0.968 |
| Blood glucose |  |  | |  |  |
| Mortality (/10,000PYs) | 5.51 | 2.91 | | 1.57 |  |
| Model 1 | 1.00 (ref.) | **0.58 (0.53, 0.63)** | | **0.37 (0.35, 0.40)** | 0.003 |
| Fully adjusted | 1.00 (ref.) | **0.62 (0.57, 0.68)** | | **0.43 (0.40, 0.47)** | 0.012 |
| Blood pressure |  |  | |  |  |
| Mortality (/10,000PYs) | 2.79 | 1.19 | | 0.93 |  |
| Model 1 | 1.00 (ref.) | **0.60 (0.56, 0.63)** | | **0.64 (0.57, 0.71)** | 0.105 |
| Fully adjusted | 1.00 (ref.) | **0.61 (0.57, 0.65)** | | **0.64 (0.58, 0.71)** | 0.127 |

Abbreviations: HR, hazard ratios; CI, confidence intervals; PYs, person-years. Model 1 adjusted age, sex, region, and ethnicity. Fully adjusted included age, sex, region, ethnicity, education level, Townsend deprivation index, household income, employ status, and alcohol consumption. HR (95% CI) in bold indicates *P*≤ 0.05.

**
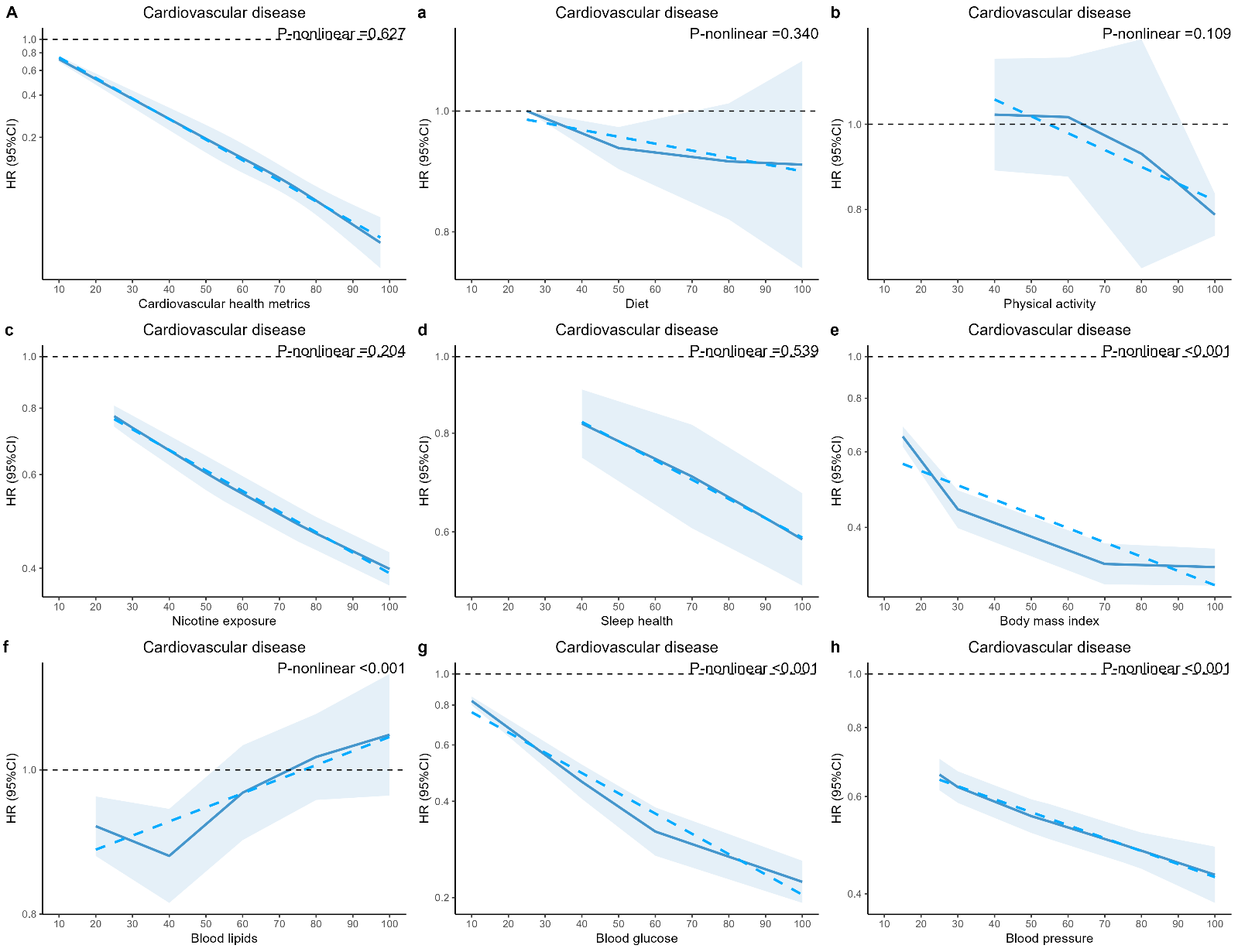
**

**Figure S2 Association between cardiovascular health metrics components and CVD mortality in cox regression with restricted cubic spline**

The hazard ratios (solid line) and 95% confidence intervals (band) were estimated by fitting restricted cubic spline Cox regression models with 5th, 35th, 65th and 95th knots, in which cardiovascular health metrics was modeled as a continuous variable. The minimum value (cardiovascular health metrics =0) was set as the reference. Cox regression model was adjusted for age, sex, region, ethnicity, education level, Townsend deprivation index, household income, employ status, and alcohol consumption.

**
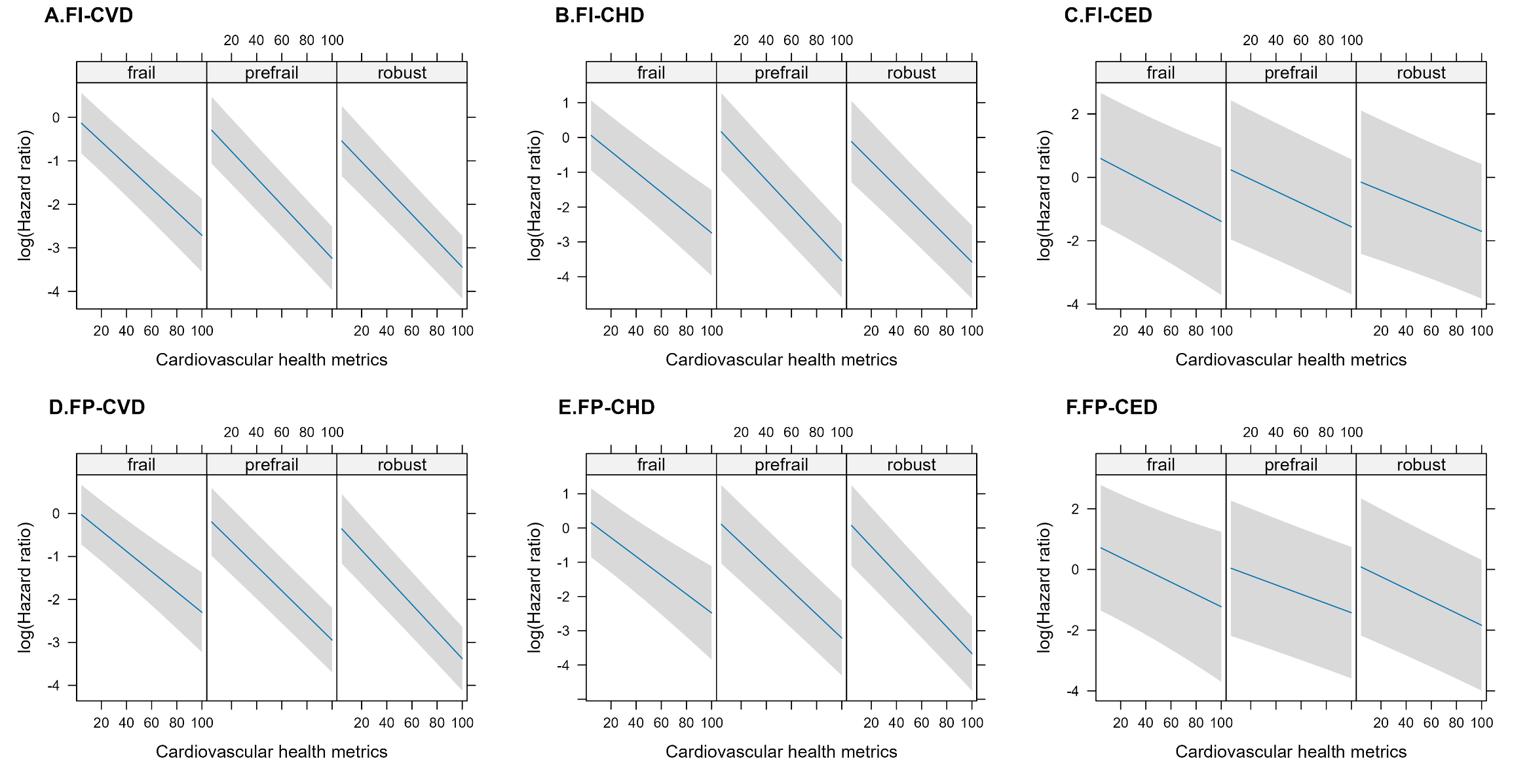
Figure S3 Interaction plots of the association of frailty status and cardiovascular health with the risk of mortality from cardiovascular and cerebrovascular disease**

FI, frailty index; FP, Fried phenotype; CVD, cardiovascular disease; CHD, coronary heart disease; CED, cerebrovascular disease. Cox regression model was adjusted for age, sex, region, ethnicity, education level, Townsend deprivation index, household income, employ status, and alcohol consumption.

**
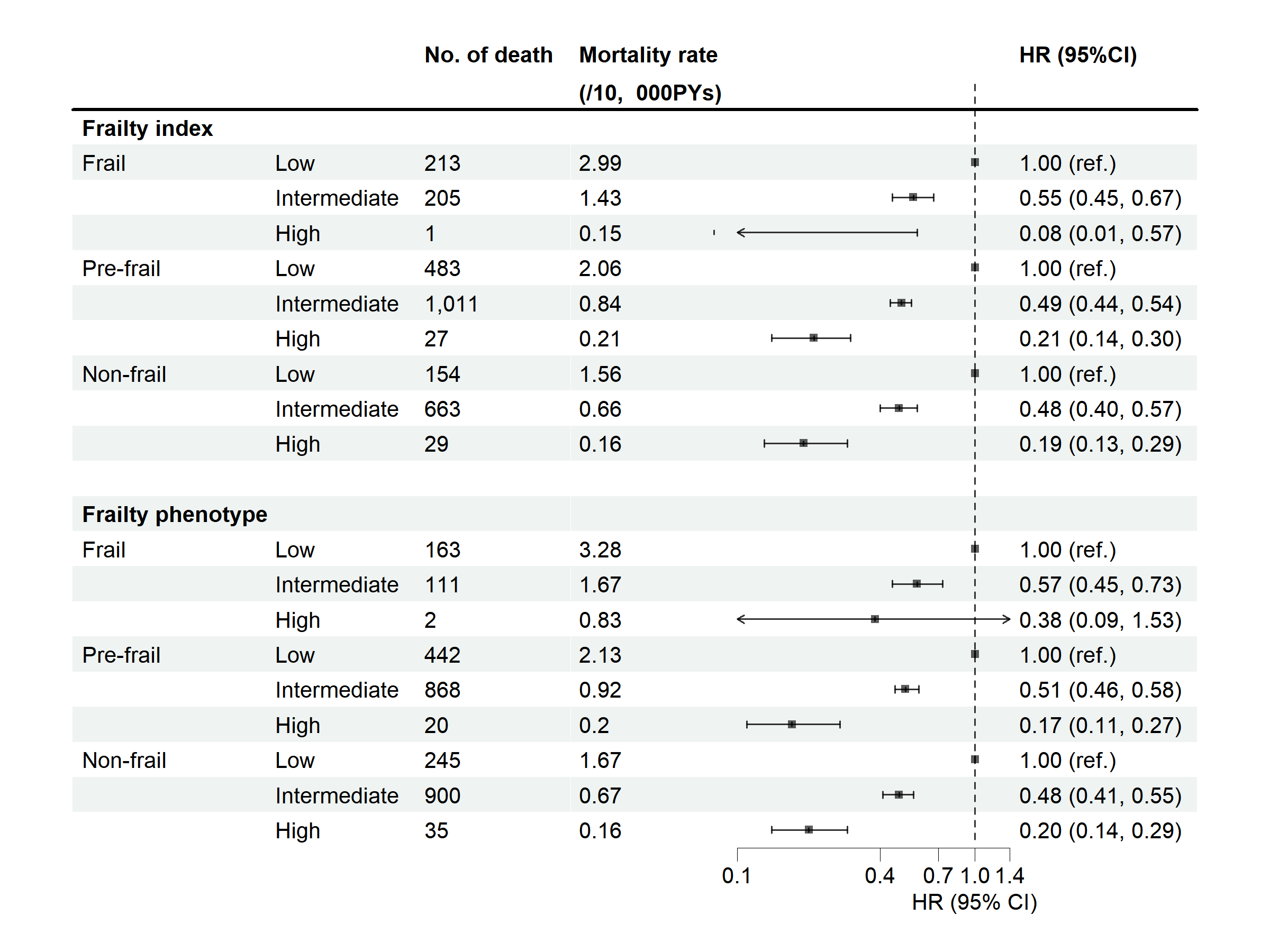
Figure S4 Association between cardiovascular health metrics and risk of CHD mortality by frailty status**

HR, hazard ratios; CI, confidence intervals; PYs, person-years. Cox regression model was adjusted for age, sex, region, ethnicity, education level, Townsend deprivation index, household income, employ status, and alcohol consumption. The P-values for multiplicative interaction of frailty index and frailty phenotype were 0.596 and 0.536, the P-values for additive interaction were 0.163 and 0.247.

**
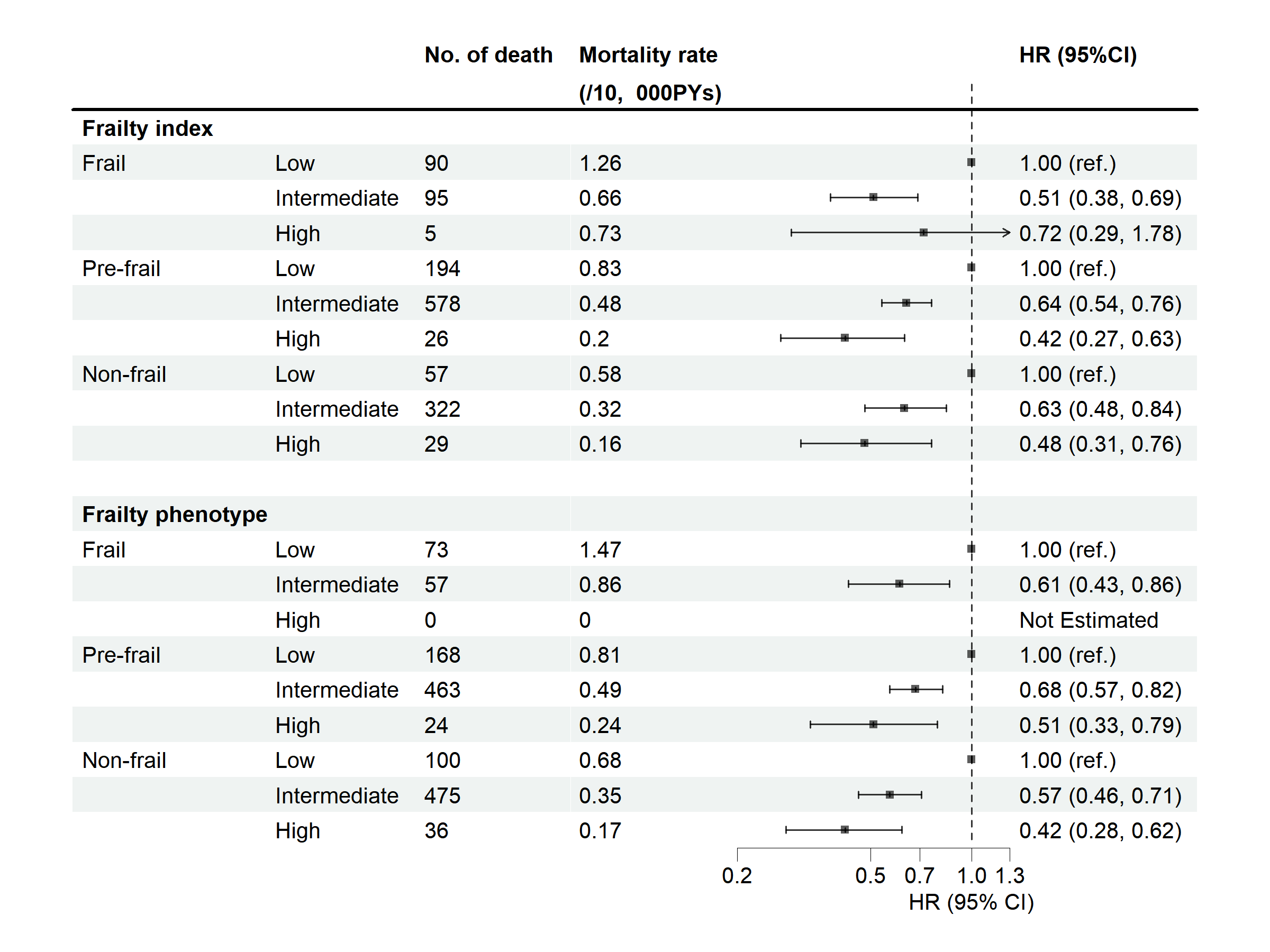
Figure S5 Association between cardiovascular health metrics and risk of cerebrovascular disease mortality by frailty status**

HR, hazard ratios; CI, confidence intervals; PYs, person-years. Cox regression model was adjusted for age, sex, region, ethnicity, education level, Townsend deprivation index, household income, employ status, and alcohol consumption. The P-values for multiplicative interaction of frailty index and frailty phenotype were 0.556 and 0.486, the P-values for additive interaction were 0.109 and 0.163.

**
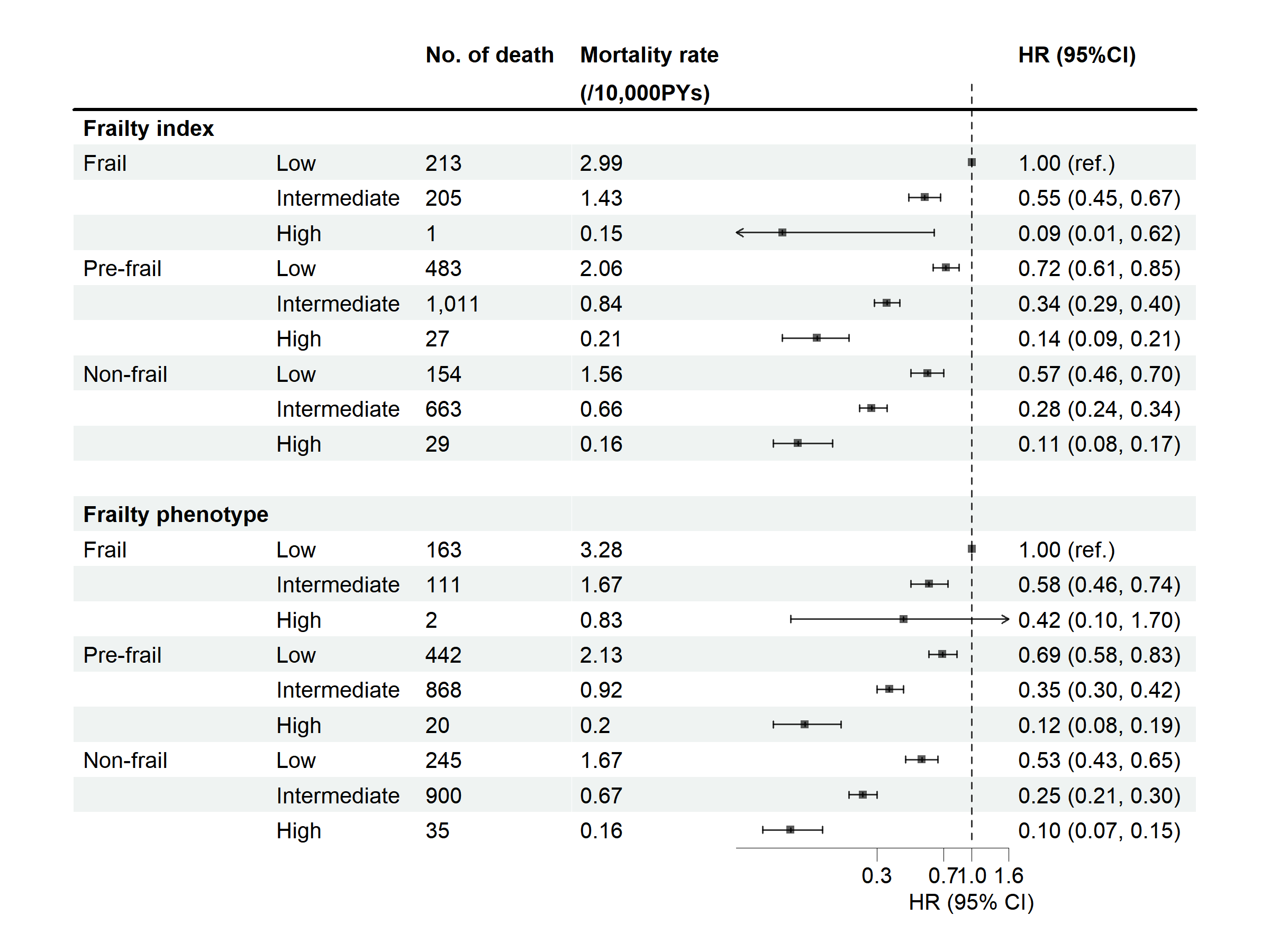
Figure S6 Joint association of frailty status and cardiovascular health metrics with mortality from CHD**

HR, hazard ratios; CI, confidence intervals; PYs, person-years. Cox regression model was adjusted for age, sex, region, ethnicity, education level, Townsend deprivation index, household income, employ status, and alcohol consumption.

**
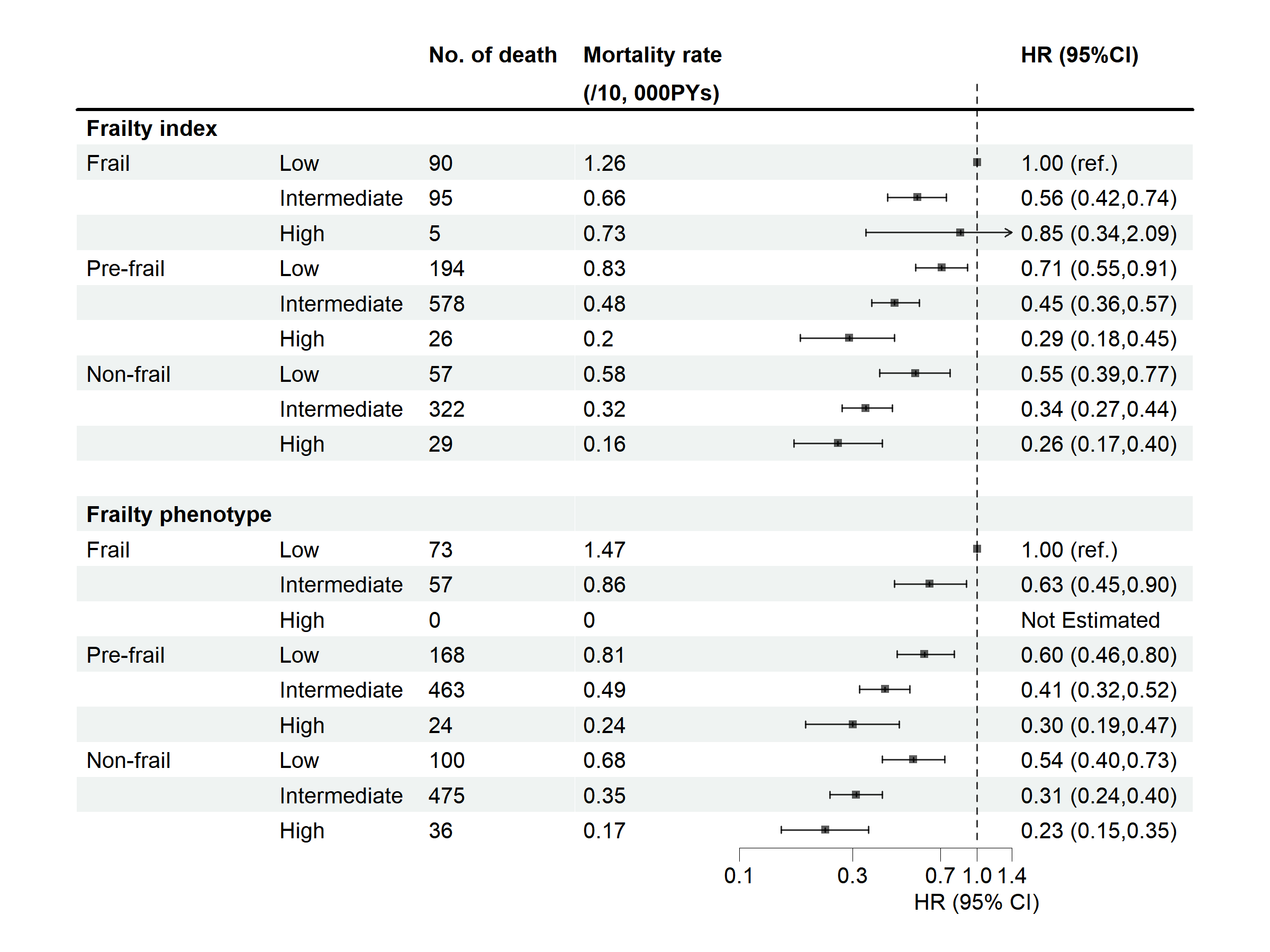
Figure S7 Joint association of frailty status and cardiovascular health metrics with mortality from cerebrovascular disease**

HR, hazard ratios; CI, confidence intervals; PYs, person-years. Cox regression model was adjusted for age, sex, region, ethnicity, education level, Townsend deprivation index, household income, employ status, and alcohol consumption.

**
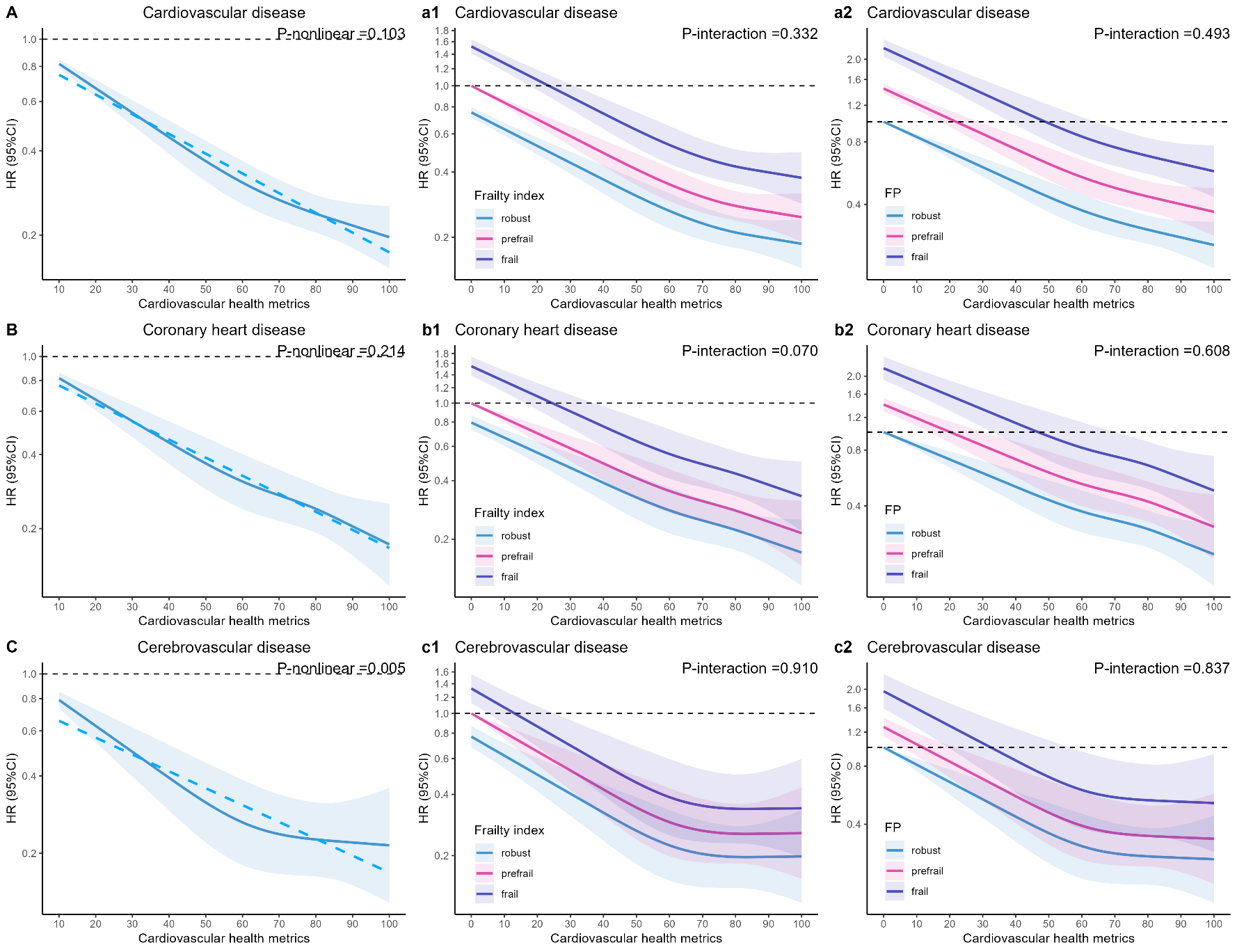
**

**Figure S8 Association between health behaviors metrics and CVD mortality by frailty status in cox regression with restricted cubic spline**

FP, frailty phenotypes. The hazard ratios (solid line) and 95% confidence intervals (band) were estimated by fitting restricted cubic spline Cox regression models with 5th, 35th, 65th and 95th knots, in which cardiovascular health metrics was modeled as a continuous variable. The minimum value (cardiovascular health metrics =0) was set as the reference. Cox regression model was adjusted for age, sex, region, ethnicity, education level, Townsend deprivation index, household income, employ status, and alcohol consumption.

**
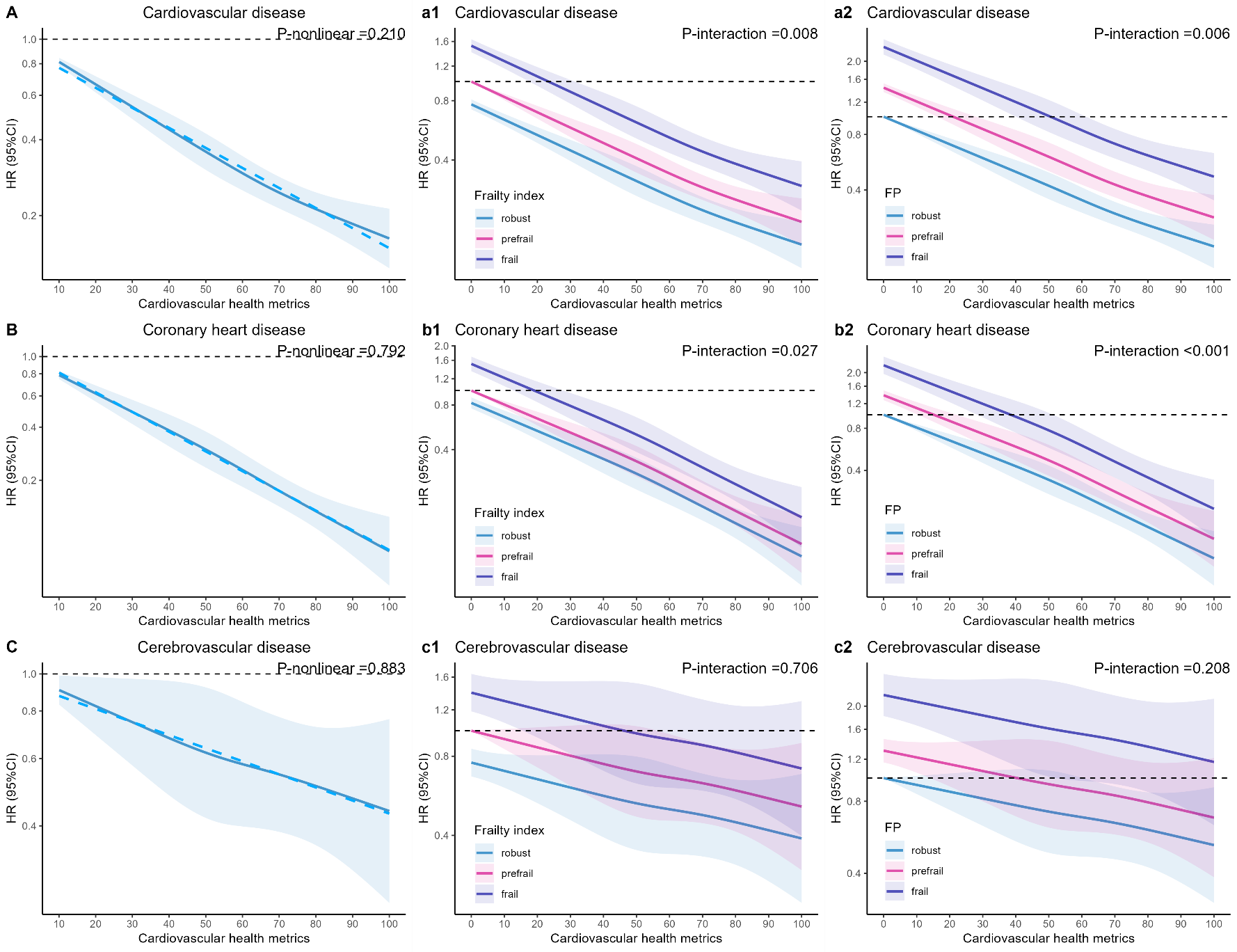
Figure S9 Association between health factors metrics and CVD mortality by frailty status in cox regression with restricted cubic spline**

FP, frailty phenotypes. The hazard ratios (solid line) and 95% confidence intervals (band) were estimated by fitting restricted cubic spline Cox regression models with 5th, 35th, 65th and 95th knots, in which cardiovascular health metrics was modeled as a continuous variable. The minimum value (cardiovascular health metrics =0) was set as the reference. Cox regression model was adjusted for age, sex, region, ethnicity, education level, Townsend deprivation index, household income, employ status, and alcohol consumption.

**
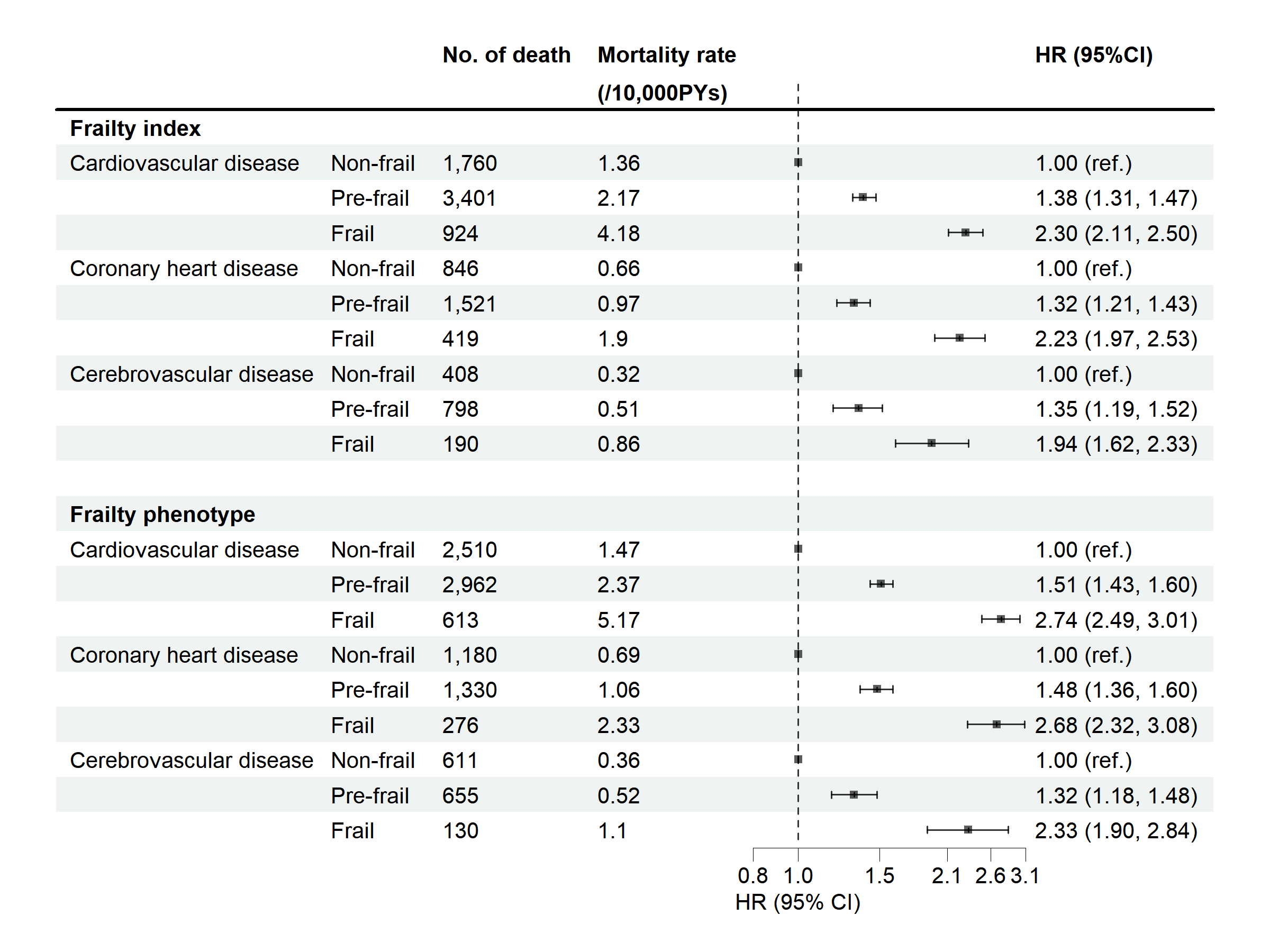
Figure S10 Association of frailty index and frailty phenotype with CVD mortality**

HR, hazard ratios; CI, confidence intervals; PYs, person-years. Cox regression model was adjusted for age, sex, region, ethnicity, education level, Townsend deprivation index, household income, employ status, and alcohol consumption.

**Table S4 Association between cardiovascular health metrics and CVD mortality by Subgroups**

|  | Cardiovascular health metrics | | | | *P* for interaction |
| --- | --- | --- | --- | --- | --- |
|  | Low (0-49) | | Moderate (50-79) | High (80-100) |  |
| Age (years) |  |  | |  | 0.001 |
| <60 | 1.00 (ref.) | 0.47 (0.42, 0.52) | | 0.17 (0.13, 0.23) |  |
| ≥60 | 1.00 (ref.) | 0.51 (0.48, 0.55) | | 0.32 (0.27, 0.39) |  |
| Gender (%) |  |  | |  | 0.029 |
| Women | 1.00 (ref.) | 0.45 (0.41, 0.50) | | 0.25 (0.20, 0.31) |  |
| Men | 1.00 (ref.) | 0.53 (0.49, 0.56) | | 0.26 (0.21, 0.32) |  |
| Ethnicity (%) |  |  | |  | 0.813 |
| White | 1.00 (ref.) | 0.50 (0.47, 0.53) | | 0.25 (0.22, 0.30) |  |
| Others | 1.00 (ref.) | 0.50 (0.39, 0.65) | | 0.21 (0.09, 0.53) |  |
| Educational level (%) |  |  | |  | 0.079 |
| College or above | 1.00 (ref.) | 0.44 (0.38, 0.50) | | 0.22 (0.17, 0.28) |  |
| Below college | 1.00 (ref.) | 0.51 (0.48, 0.55) | | 0.27 (0.22, 0.33) |  |
| Townsend deprivation index |  |  | |  | 0.897 |
| Above median value | 1.00 (ref.) | 0.50 (0.47, 0.55) | | 0.26 (0.20, 0.33) |  |
| Below median value | 1.00 (ref.) | 0.48 (0.44, 0.53) | | 0.24 (0.20, 0.30) |  |
| Alcohol intake frequency (%) |  |  | |  | 0.256 |
| Never drinker | 1.00 (ref.) | 0.53 (0.43, 0.65) | | 0.31 (0.19, 0.53) |  |
| Drinker | 1.00 (ref.) | 0.66 (0.61, 0.71) | | 0.39 (0.32, 0.47) |  |
| No. of long-term conditions |  |  | |  | 0.358 |
| None | 1.00 (ref.) | 0.52 (0.45, 0.61) | | 0.28 (0.21, 0.37) |  |
| One | 1.00 (ref.) | 0.53 (0.49, 0.57) | | 0.29 (0.24, 0.36) |  |
| Two or more | 1.00 (ref.) | 0.55 (0.49, 0.62) | | 0.32 (0.20, 0.51) |  |
| Chronic medication status (%) |  |  | |  |  |
| Normal | 1.00 (ref.) | 0.51 (0.47, 0.55) | | 0.27 (0.22, 0.32) | 0.024 |
| Polypharmacy | 1.00 (ref.) | 0.58 (0.53, 0.64) | | 0.33 (0.23, 0.46) |  |

Abbreviations: Cox regression model was adjusted for age, sex, region, ethnicity, education level, Townsend deprivation index, household income, employ status, and alcohol consumption, excluding the stratification factors corresponding to each model.

**
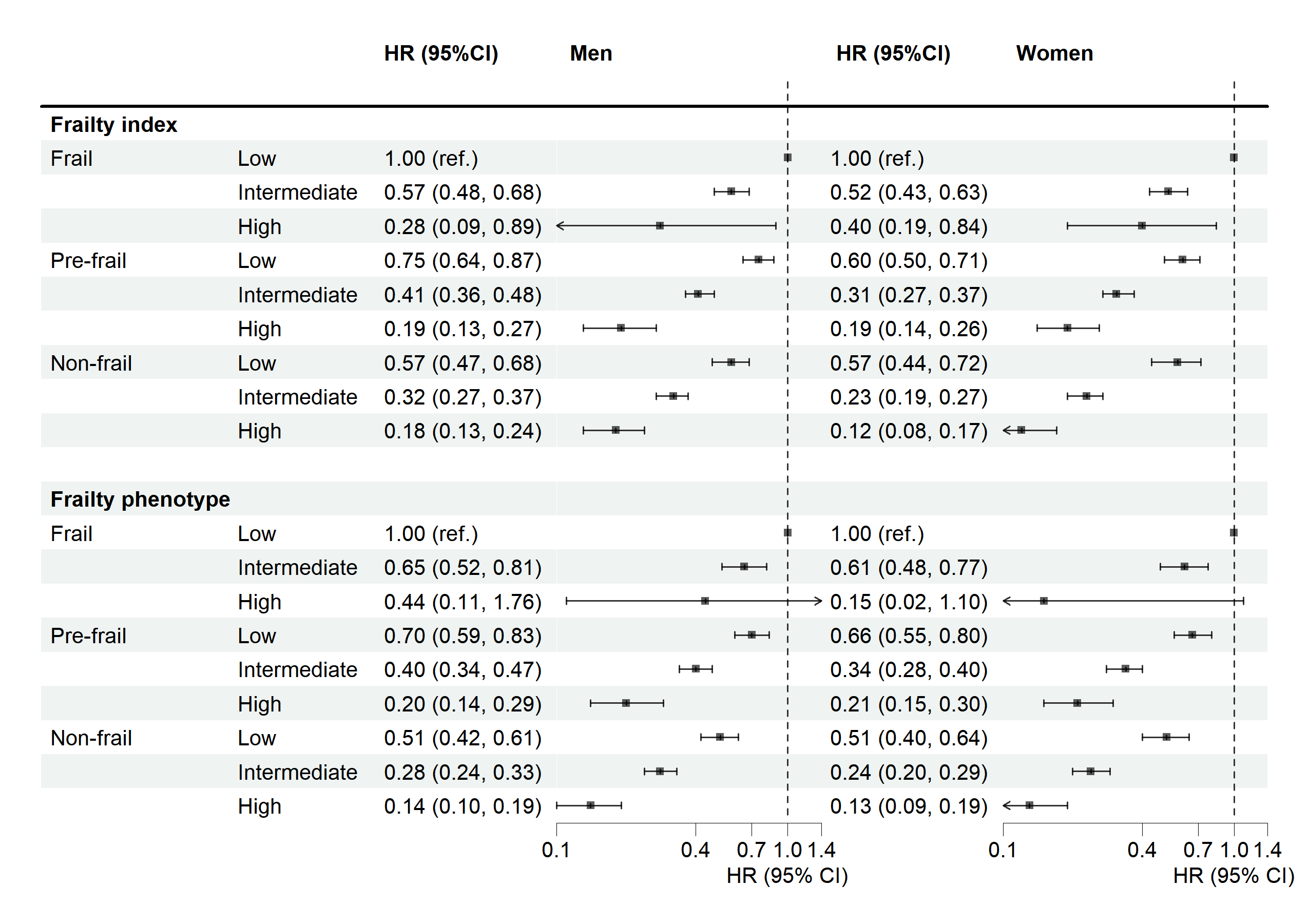
F****igure S11 Joint association of frailty status and cardiovascular health metrics with risk of CVD mortality by gender**

HR, hazard ratios; CI, confidence intervals; PYs, person-years. Cox regression model was adjusted for age, region, ethnicity, education level, Townsend deprivation index, household income, employ status, and alcohol consumption. The P-values for multiplicative interaction of frailty index and frailty phenotype were 0.032 and 0.495.


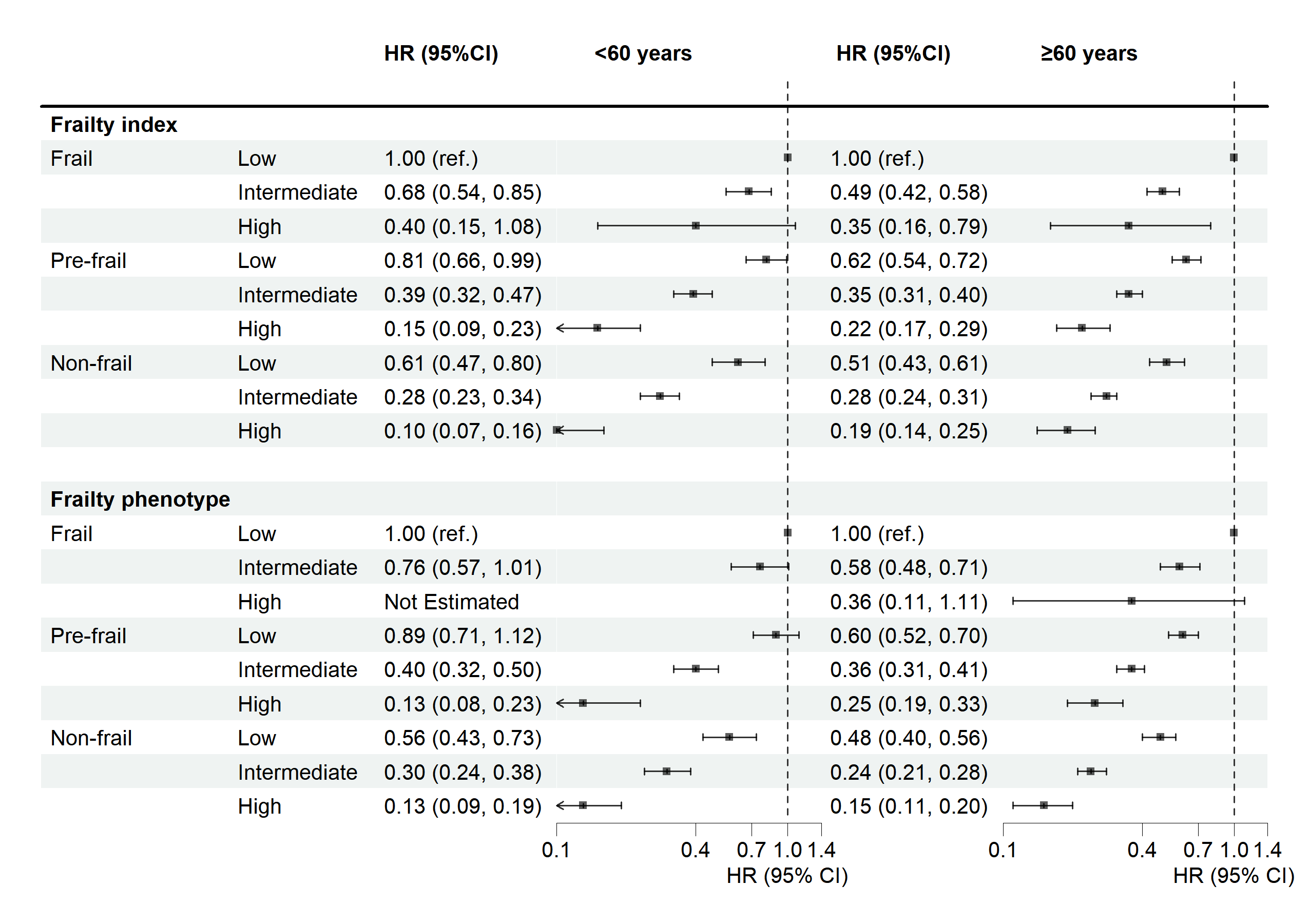


**Figure S12 Joint association of frailty status and cardiovascular health metrics with risk of CVD mortality by age**

HR, hazard ratios; CI, confidence intervals; PYs, person-years. Cox regression model was adjusted for gender, region, ethnicity, education level, Townsend deprivation index, household income, employ status, and alcohol consumption. The P-values for multiplicative interaction of frailty index and frailty phenotype were both <0.001.

**
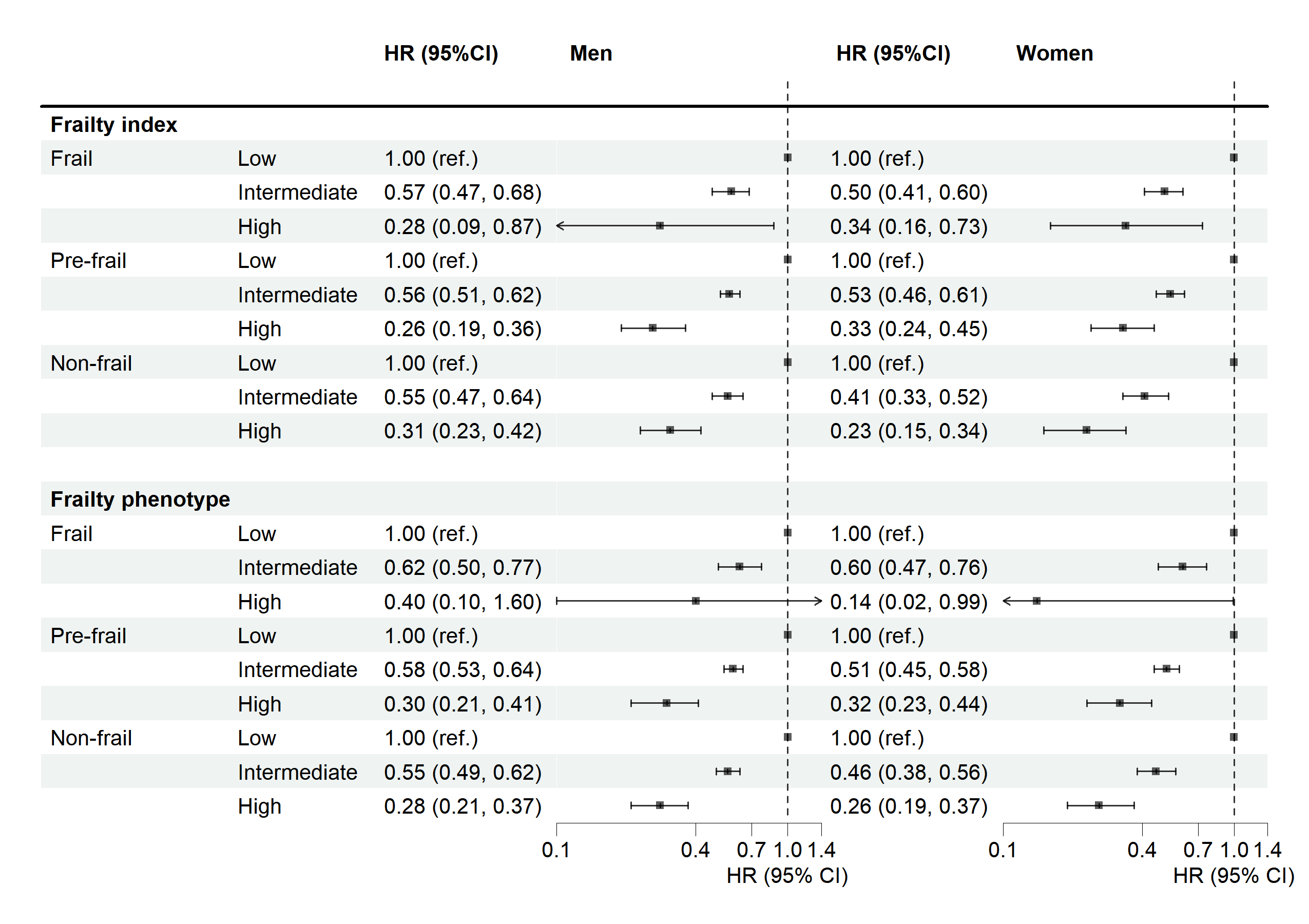
Figure S13 Association between cardiovascular health indicators and risk of cardiovascular disease mortality by frailty status with gender stratification**

HR, hazard ratios; CI, confidence intervals; PYs, person-years. Cox regression model was adjusted for age, region, ethnicity, education level, Townsend deprivation index, household income, employ status, and alcohol consumption. The P-values for multiplicative interaction of frailty index and frailty phenotype were 0.032 and 0.495.

**
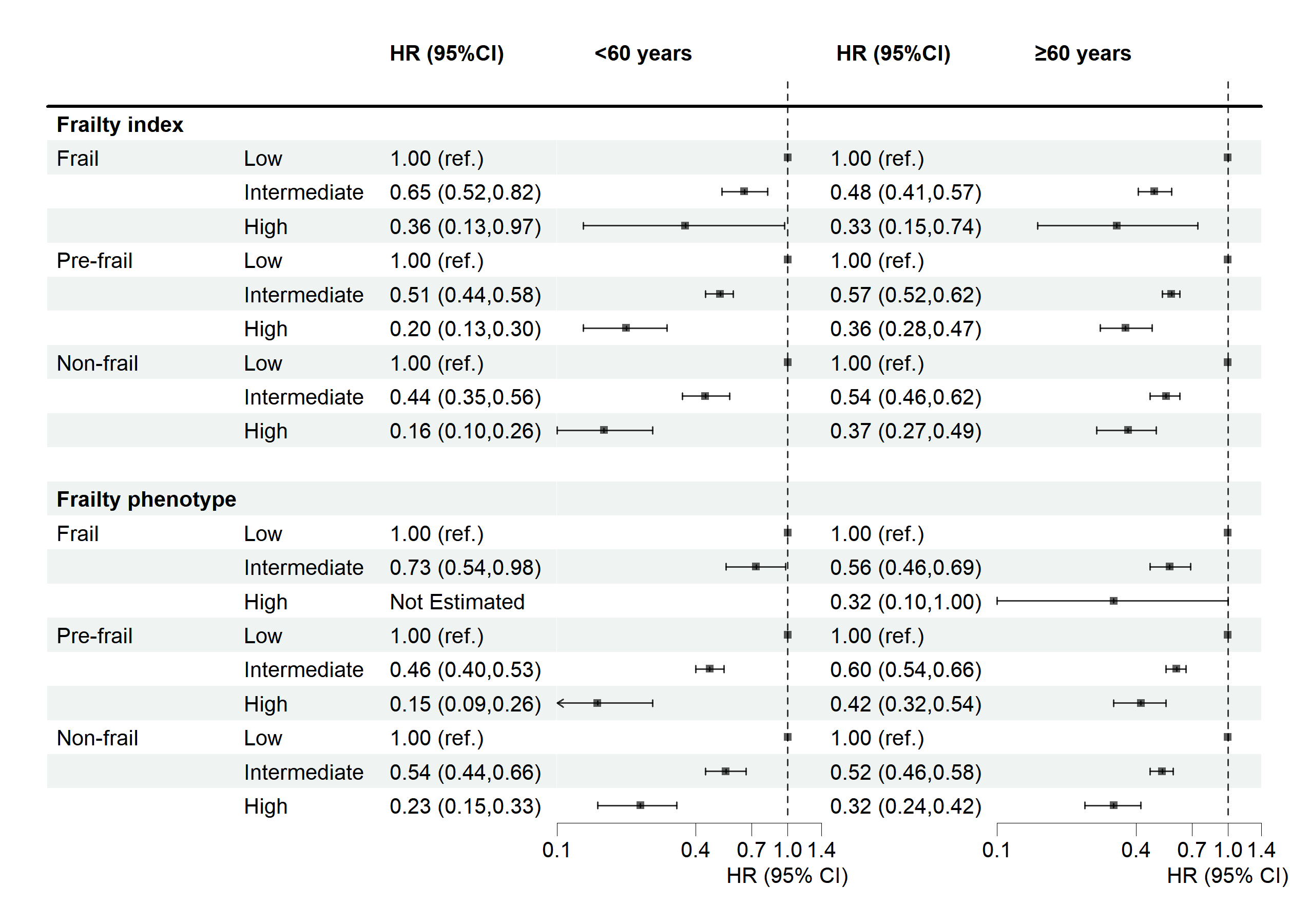
Figure S14 Association between cardiovascular health indicators and risk of cardiovascular disease mortality by frailty status with age stratification**

HR, hazard ratios; CI, confidence intervals; PYs, person-years. Cox regression model was adjusted for gender, region, ethnicity, education level, Townsend deprivation index, household income, employ status, and alcohol consumption. The P-values for multiplicative interaction of frailty index and frailty phenotype were both <0.001.

**
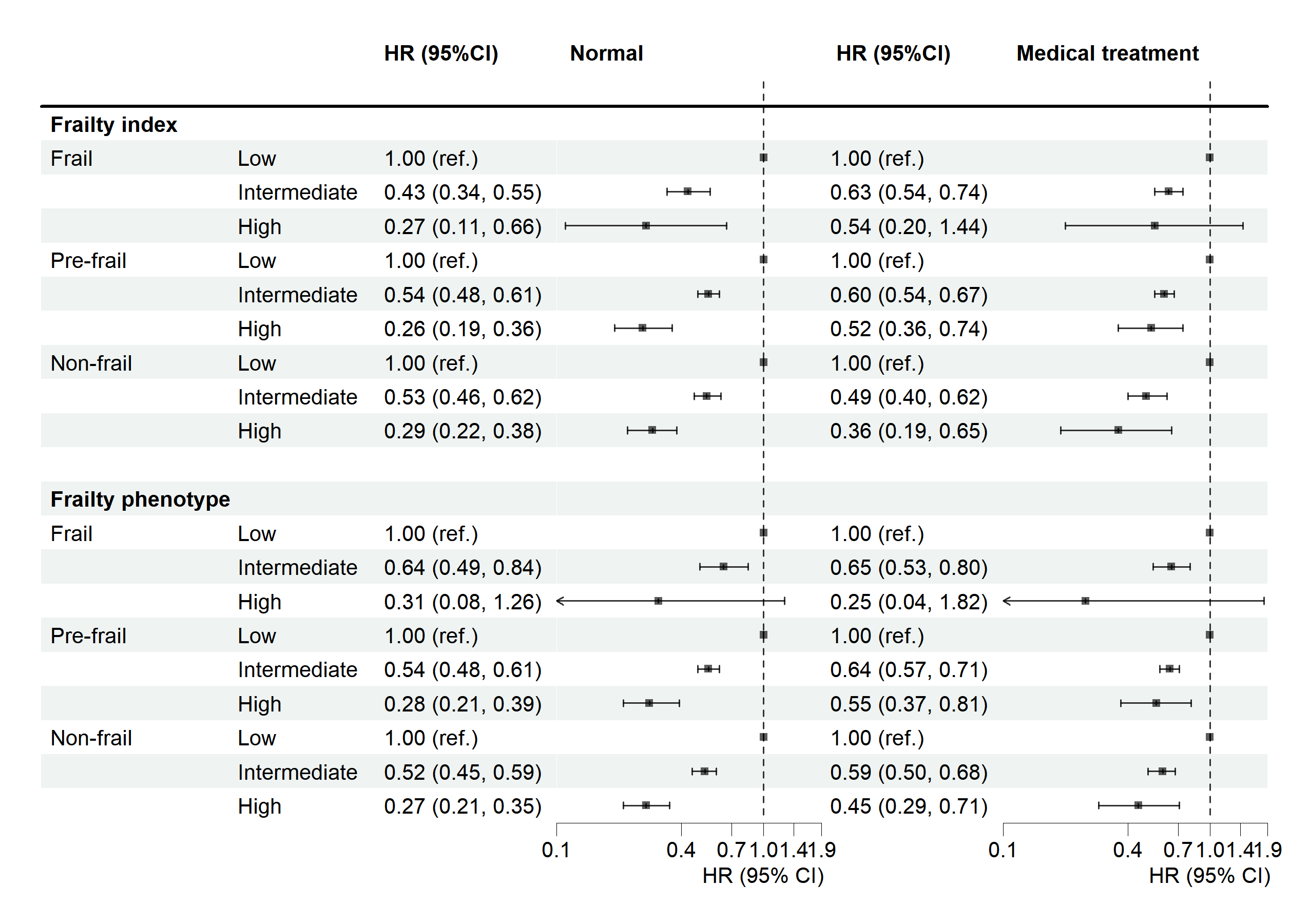
Figure S15 Association between cardiovascular health indicators and risk of cardiovascular disease mortality by frailty status with medical treatment of blood pressure, cholesterol and diabetes stratification**

HR, hazard ratios; CI, confidence intervals; PYs, person-years. Cox regression model was adjusted for gender, region, ethnicity, education level, Townsend deprivation index, household income, employ status, and alcohol consumption. The P-values for multiplicative interaction of frailty index and frailty phenotype were 0.005 and 0.009.

**
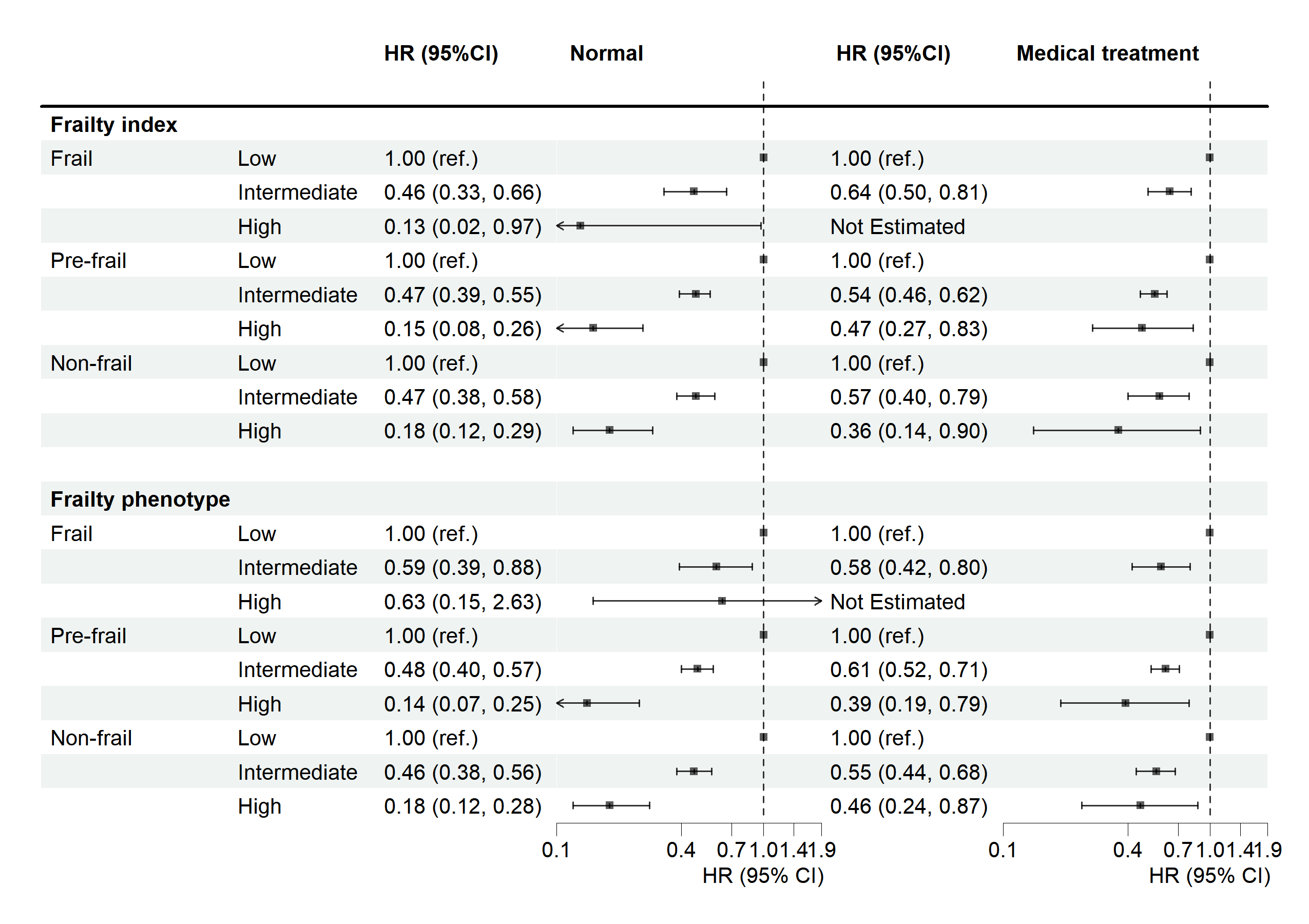
Figure S16 Association between cardiovascular health indicators and risk of CHD mortality by frailty status with medical treatment of blood pressure, cholesterol and diabetes stratification**

HR, hazard ratios; CI, confidence intervals; PYs, person-years. Cox regression model was adjusted for gender, region, ethnicity, education level, Townsend deprivation index, household income, employ status, and alcohol consumption. The P-values for multiplicative interaction of frailty index and frailty phenotype were 0.033 and 0.058.

**
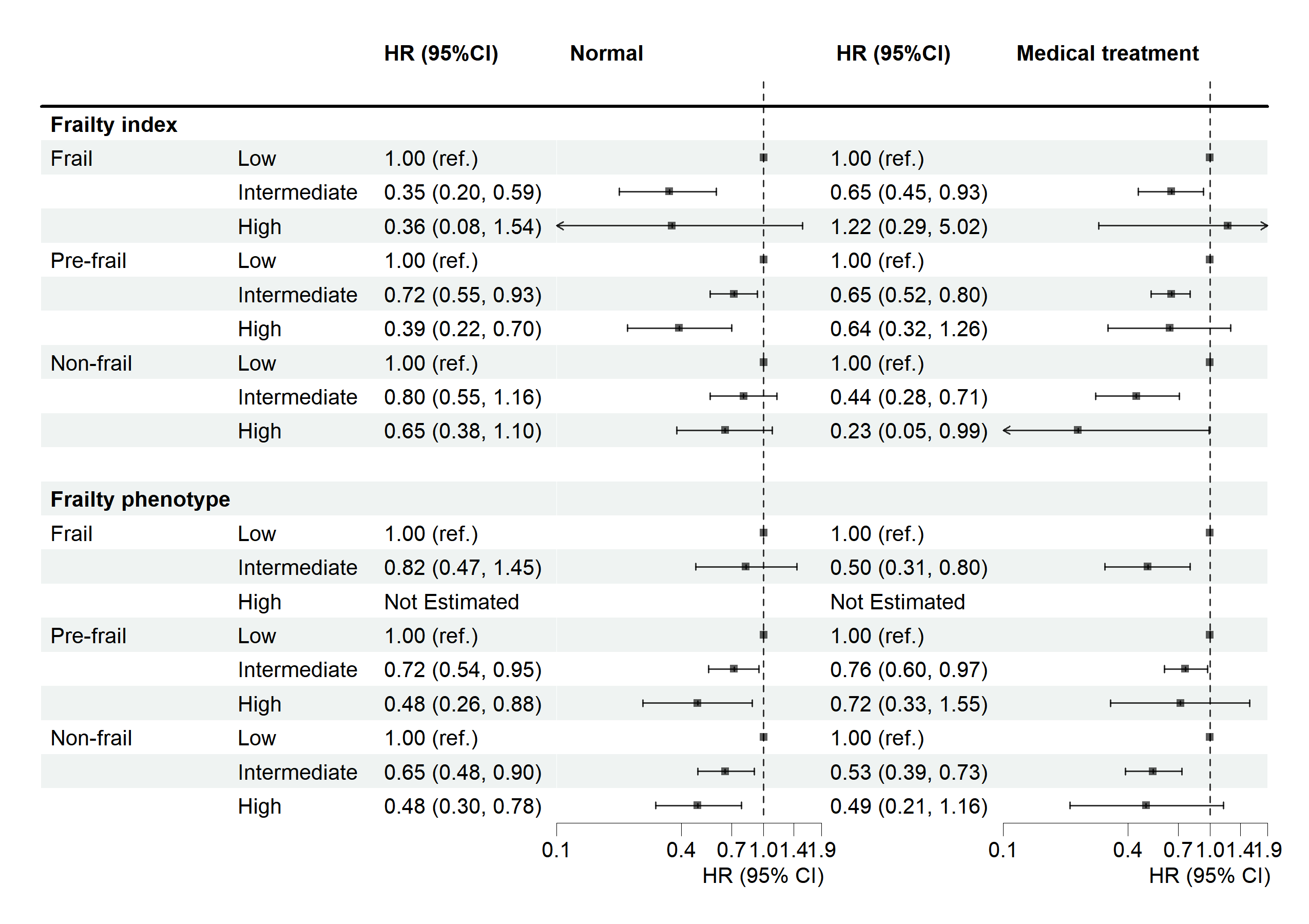
Figure S17 Association between cardiovascular health indicators and risk of cerebrovascular disease mortality by frailty status with medical treatment of blood pressure, cholesterol and diabetes stratification**

HR, hazard ratios; CI, confidence intervals; PYs, person-years. Cox regression model was adjusted for gender, region, ethnicity, education level, Townsend deprivation index, household income, employ status, and alcohol consumption. The P-values for multiplicative interaction of frailty index and frailty phenotype were 0.333 and 0.269.

**
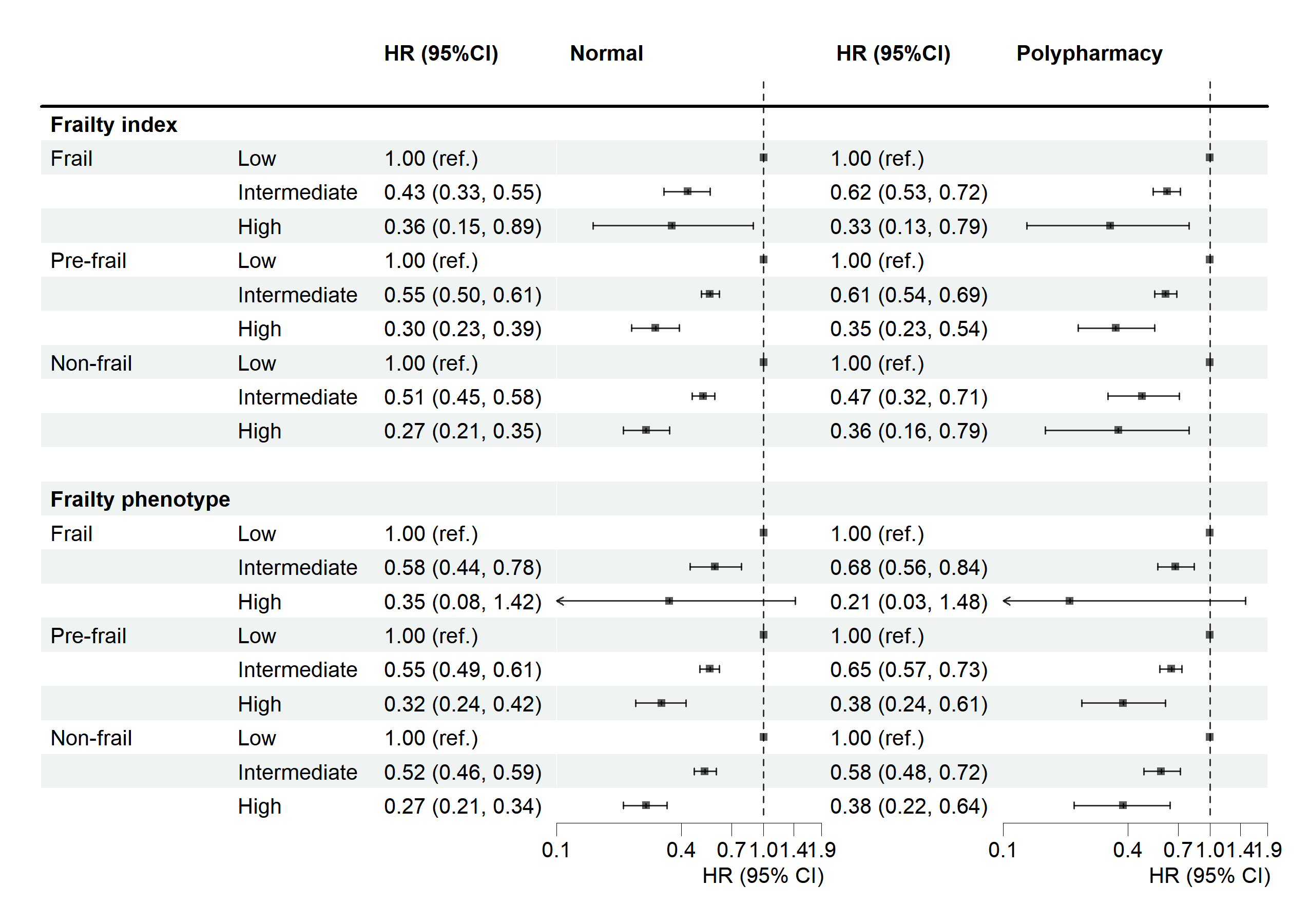
Figure S18 Association between cardiovascular health indicators and risk of cardiovascular disease mortality by frailty status with polypharmacy stratification**

HR, hazard ratios; CI, confidence intervals; PYs, person-years. Cox regression model was adjusted for gender, region, ethnicity, education level, Townsend deprivation index, household income, employ status, and alcohol consumption. The P-values for multiplicative interaction of frailty index and frailty phenotypes were 0.122 and 0.129.

**
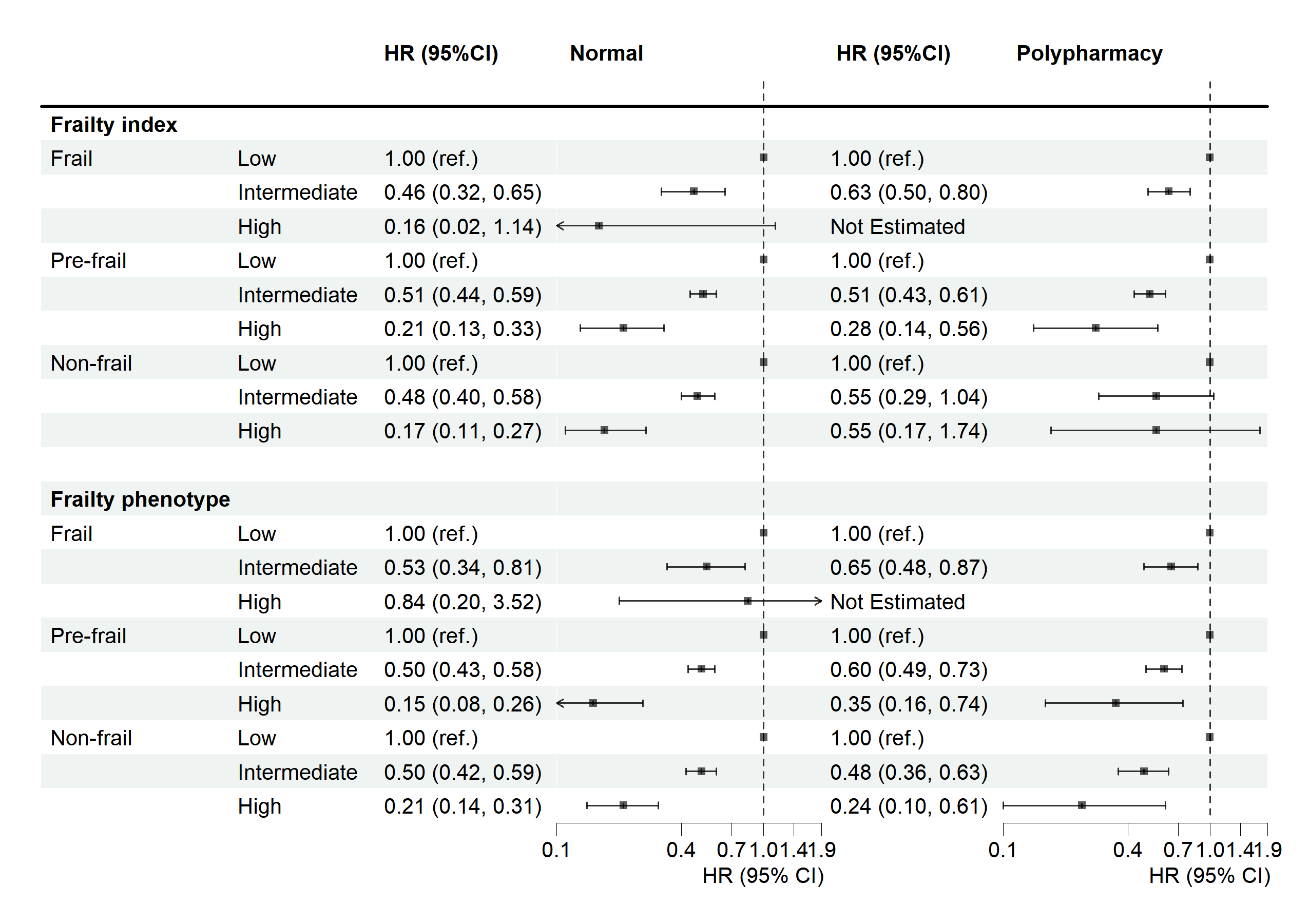
Figure S19 Association between cardiovascular health indicators and risk of CHD mortality by frailty status with polypharmacy stratification**

HR, hazard ratios; CI, confidence intervals; PYs, person-years. Cox regression model was adjusted for gender, region, ethnicity, education level, Townsend deprivation index, household income, employ status, and alcohol consumption. The P-values for multiplicative interaction of frailty index and frailty phenotype were 0.092 and 0.288.

**
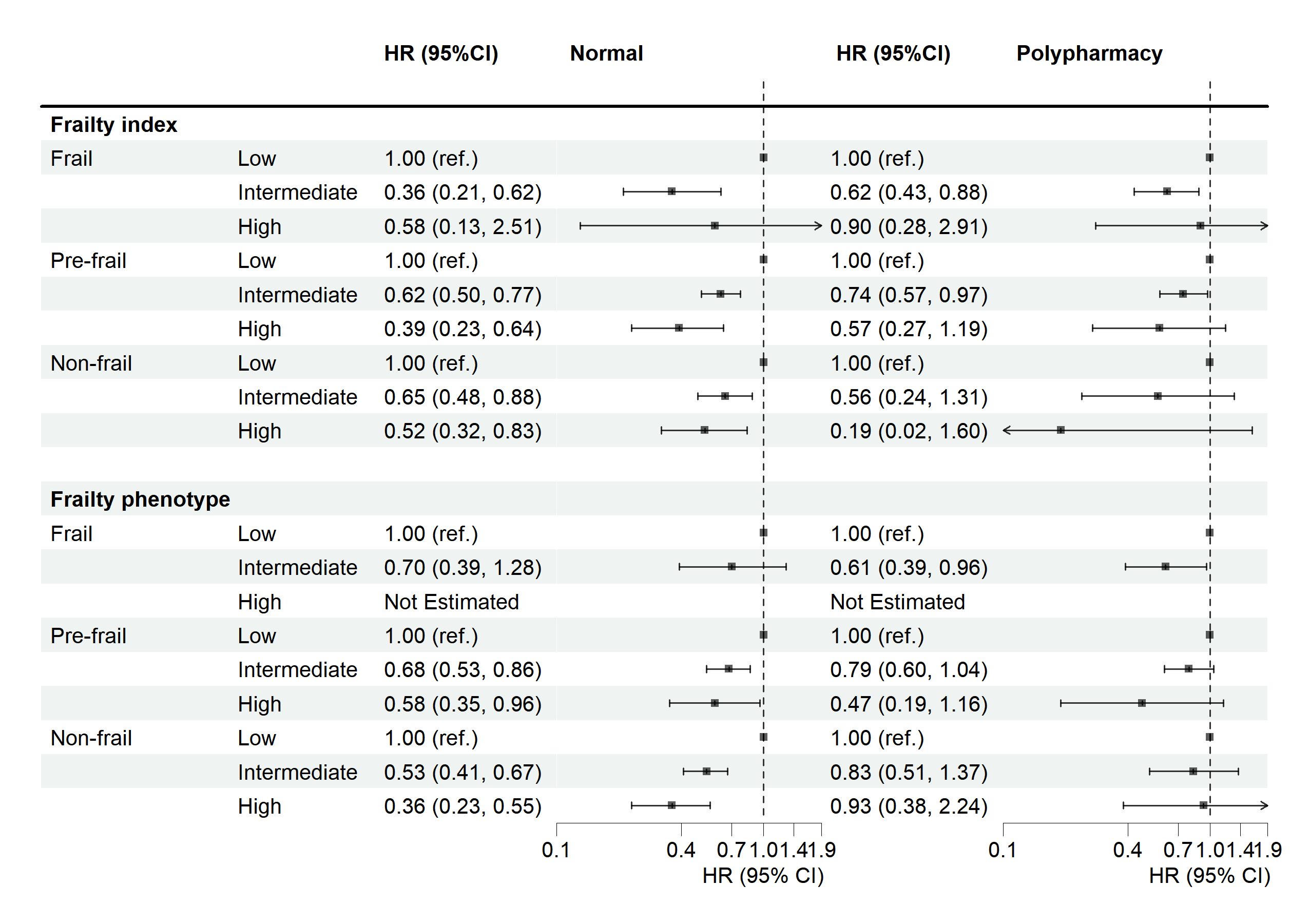
Figure S20 Association between cardiovascular health indicators and risk of cerebrovascular disease mortality by frailty status with polypharmacy stratification**

HR, hazard ratios; CI, confidence intervals; PYs, person-years. Cox regression model was adjusted for gender, region, ethnicity, education level, Townsend deprivation index, household income, employ status, and alcohol consumption. The P-values for multiplicative interaction of frailty index and frailty phenotype were 0.691 and 0.206.

**Table S5 Association of cardiovascular health metrics with CVD mortality adjusted for different medical treatments**

|  | Cardiovascular health metrics | | |
| --- | --- | --- | --- |
|  | Low (0-49) | Moderate (50-79) | High (80-100) |
| Model 3 (HR (95% CI)) | 1.00 (ref.) | 0.50 (0.47, 0.53) | 0.25 (0.22, 0.29) |
| Model 3+adjusted for BP medication | 1.00 (ref.) | 0.50 (0.47, 0.53) | 0.25 (0.22, 0.29) |
| Model 3+adjusted for cholesterol medication | 1.00 (ref.) | 0.57 (0.53, 0.62) | 0.43 (0.33, 0.57) |
| Model 3+adjusted for diabetes medication | 1.00 (ref.) | 0.55 (0.51, 0.60) | 0.37 (0.28, 0.48) |
| Model 3+adjusted for BP, cholesterol and diabetes medication | 1.00 (ref.) | 0.57 (0.52, 0.61) | 0.39 (0.29, 0.51) |
| Model 3+adjusted for anti-inflammatory painkillers | 1.00 (ref.) | 0.53 (0.50, 0.57) | 0.29 (0.25, 0.34) |
| Model 3+adjusted for blood glucose medication | 1.00 (ref.) | 0.51 (0.48, 0.54) | 0.26 (0.22, 0.31) |
| Model 3+adjusted for vitamin and mineral supplements | 1.00 (ref.) | 0.50 (0.47, 0.53) | 0.25 (0.22, 0.29) |
| Model 3+adjusted for minerals and other dietary supplements | 1.00 (ref.) | 0.50 (0.47, 0.53) | 0.26 (0.22, 0.30) |
| Model 3+adjusted for polypharmacy | 1.00 (ref.) | 0.54 (0.51, 0.57) | 0.28 (0.24, 0.33) |

Abbreviations: HR, hazard ratio. CI, confidence interval. CVD, cardiovascular disease. BP, blood pressure. Cox regression model 3 was adjusted for age, sex, region, ethnicity, education level, Townsend deprivation index, household income, employ status, and alcohol consumption.

**Table S6 Sensitivity analyses of association of cardiovascular health metrics with CVD mortality**

|  | Cardiovascular health metrics | | |
| --- | --- | --- | --- |
|  | Low (0-49) | Moderate (50-79) | High (80-100) |
| Further Adjustment for long-term condition^a^ | 1.00 (ref.) | 0.54 (0.51, 0.58) | 0.30 (0.26, 0.35) |
| Excluding outcomes occurred during the first year of follow-up | 1.00 (ref.) | 0.54 (0.51, 0.56) | 0.31 (0.27, 0.37) |
| Excluding participants with cancer at baseline^b^ | 1.00 (ref.) | 0.52 (0.50, 0.55) | 0.31 (0.26, 0.36) |
| Fine & Gray Models for Competing Risk^c^ | 1.00 (ref.) | 0.44 (0.42, 0.47) | 0.19 (0.16, 0.22) |
| Time-stratified cox regression^d^ | 1.00 (ref.) | 0.50 (0.47, 0.53) | 0.26 (0.22, 0.30) |

Abbreviations: HR, hazard ratio. CI, confidence interval. CVD, cardiovascular disease. Cox regression model was adjusted for age, sex, region, ethnicity, education level, Townsend deprivation index, household income, employ status, and alcohol consumption.

^a^The number of long-term conditions was further adjusted in this sensitivity analysis.

^b^Cancer was not adjusted in this sensitivity analysis.

^c^The model was unadjusted.

^d^The model stratifies survival time by adding the interaction terms of age and gender with time grouping to the model.
